# Ancient Great Wall building materials reveal environmental changes associated with oases in northwestern China

**DOI:** 10.1038/s41598-022-27071-4

**Published:** 2022-12-29

**Authors:** Robert Patalano, Jing Hu, Qin Leng, Weiguo Liu, Huanye Wang, Patrick Roberts, Michael Storozum, Lin Yang, Hong Yang

**Affiliations:** 1 Department of Archaeology, Max Planck Institute for Geoanthropology, 07745 Jena, Germany; 2 Laboratory for Terrestrial Environments, Department of Biological and Biomedical Sciences, School of Health and Behavioral Sciences, Bryant University, Smithfield, 02917 USA; 3 State Key Laboratory of Loess and Quaternary Geology, Institute of Earth Environment, Chinese Academy of Sciences, Xi’an, 710061 China; 4 University of Chinese Academy of Sciences, Beijing, 100049 China; 5 CAS Center for Excellence in Quaternary Science and Global Change, Xi’an, 710061 China; 6 IsoTROPIC Research Group, Max Planck Institute for Geoanthropology, 07745 Jena, Germany; 7 School of Social Science, The University of Queensland, Brisbane, Australia; 8 Archaeological Studies Program, University of Philippines, Diliman, Quezon City, Philippines; 9 School of History, Classics and Archaeology, Newcastle University, Newcastle Upon Tyne, England UK; 10 National Museum of China, Beijing, 100006 China

**Keywords:** Geochemistry, Climate-change impacts, Biogeochemistry, Anthropology, Archaeology

## Abstract

Plant materials used in the construction of segments and beacon towers of the ancient Great Wall in northwestern China contain untapped potential for revealing local paleoclimatic and environmental conditions. For the first time, we characterize the molecular preservation and stable carbon and nitrogen isotope compositions of AMS-dated common reeds (*Phragmites*) collected from ancient Great Wall fascines in today’s Gansu and Xinjiang using a combination of chromatographic techniques and isotope analyses. Our molecular data, along with Scanning Electron Microscopy, demonstrate excellent preservation of these ancient reeds, which were harvested from nearby habitats during periods of significant expansion of Imperial China when climate conditions sustained sizeable oases in the region. Stable isotope data capture differential rates of environmental change along the eastern margin of the Tarim Basin since the Han Dynasty (170 BC), implying that significant surface-water hydrological changes occurred only after the Song Dynasty (1160 AD) due to regional climate change. This study reveals the wealth of environmental and climate information obtainable from these site-specific organic building materials and establishes the foundation for further applications of advanced molecular, biochemical, and isotopic technologies to study these common and widely-distributed organic archaeological materials.

## Introduction

**Figure 1:**
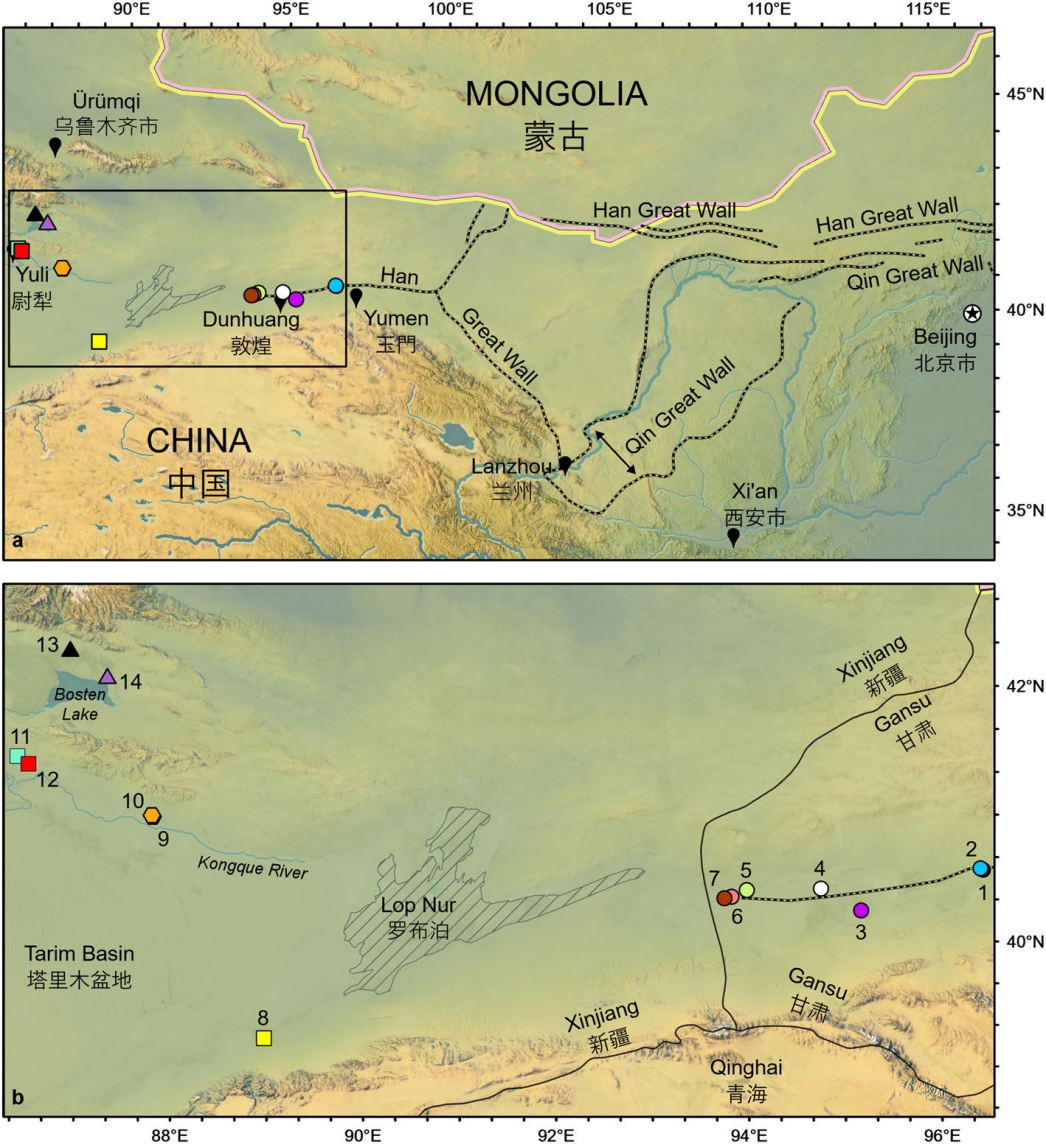
Location of the study area in northwestern China. (**a**) Extent of Qin and Han Great Wall segments across northern China. (**b**) Site locations in Gansu and Xinjiang: (1) Han Dynasty Great Wall segment; (2) Beacon tower near Site 1; (3) Beacon tower near Guazhou town; (4) Xijiandun Beacon Tower; (5) Cang Ting Sui Beacon Tower at Yumenguan; (6) Great Wall Heritage Site; (7) Majuanwan Great Wall segments; (8) Milan Castle Heritage Site; (9) Buddha Tower, south end of the Yingpan City Heritage Site; (10) City wall, north end of the Yingpan City Heritage Site; (11) Yakelun Beacon Tower; (12) Sunji Beacon Tower; (13) Tahaqi Beacon Tower; (14) Sishilidadun Beacon Tower. Circles date to the Han Dynasty, hexagons to the Jin Dynasty, squares to the Tang Dynasty, and triangles to the Song Dynasty. Maps were created using ArcGIS Pro desktop GIS software developed by Esri.

**Figure 2:**
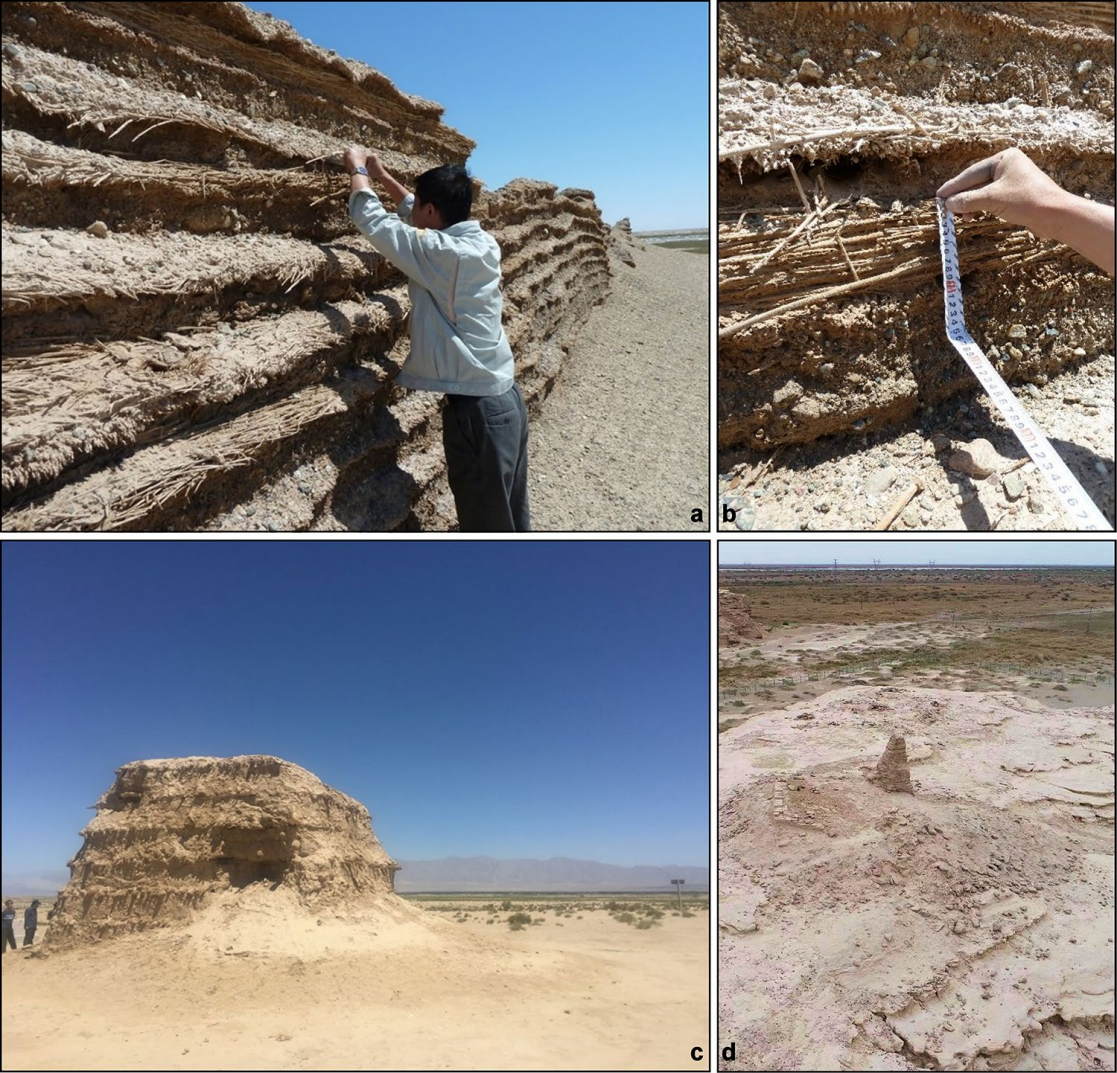
Remnant wall segments and beacon towers of northwestern China. (**a**) Sampling of *Phragmites* culms from a wall section at Majuanwan (Site 7); (**b**) Reed fascines alternating with layers of rammed earth at Majuanwan. Near center is a fascine of *Phragmites* culms that were sampled for analysis; (**c**) Remnants of Sishilidadun Beacon Tower (Site 14) dating to the Song Dynasty. Although not visible, the beacon tower was constructed like the wall section in panel **a**, with rammed earth alternating with reed fascines; (**d**) Low altitude air photo of the Yakelun Beacon Tower (Site 11), courtesy of Xingjun Hu. Note the wild plants growing adjacent to the tower.

As one of the most recognizable world heritage sites, the Great Wall of China is a manifestation of the engineering capabilities and architectural achievements of multiple Chinese dynasties^1^. What is perhaps less well known, however, is that the iconic brick and stone walls built during the Ming Dynasty in the 15th century AD^2^, are only part of a series of multi-material fortifications that stretch across northern China from Hebei Province to Xinjiang Uyghur Autonomous Region (Fig. 1, Data S1)^1,3–7^. Walls and fortresses dating back to as early as the Warring States period (475–221 BC), were constructed using locally available materials, with reed fascines and wood bundles interbedded with gravel-mixed rammed earth (Fig. 2). Following the unification of China in 221 BC, an array of fascine and rammed-earth ramparts was established to protect against the powerful northern Xiongnu and Xianbei states^8,9^, and then in the early 2nd Century BC, these border defenses became essential in expanding the territories of the Han Dynasty from the central Chinese plains into the western frontier (including today’s Xinjiang and Gansu Province)^4,10^.

While remnants of the Han and later dynasty walls along the Shule River in Gansu and Inner Mongolia Autonomous Region have been surveyed^11–14^, isolated beacon towers (Fig. 2c) along the Kongque River (Fig. 1b) in Xinjiang are still relatively unknown despite being described in historical texts like the 5th Century AD “Book of Later Han”^9^. Nevertheless, recent surveys in Gansu and Xinjiang have identified new and well-preserved remnants of the ancient Great Wall in what was once the western reach of Imperial China^15^. These fortifications served as a military communication and warning system, symbolic political borders, and rest stops for merchants traveling along the ancient Silk Road^4,6,7,16^.

**Figure 3:**
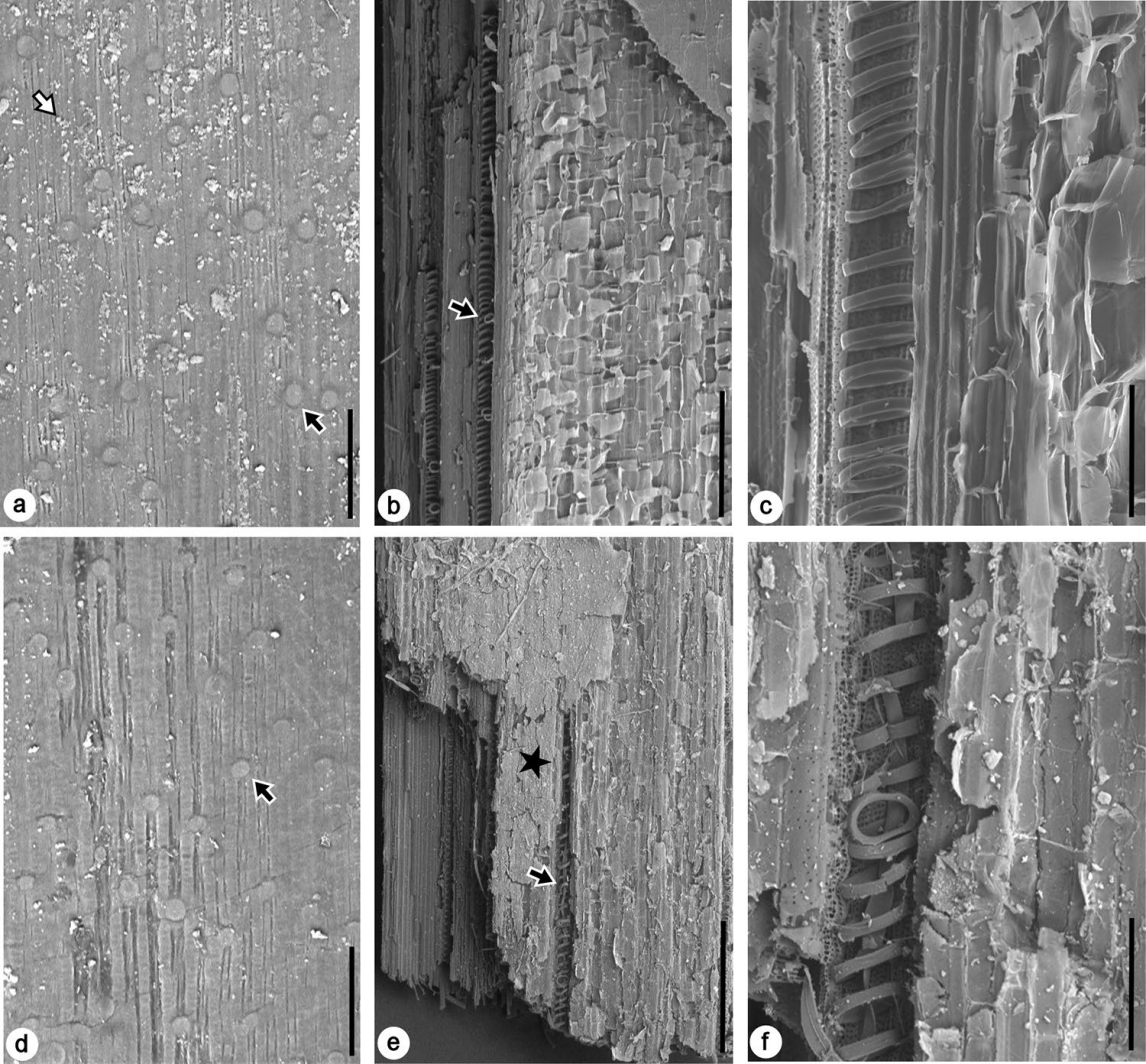
SEM photographs of modern (*Phragmites australis*) and ancient (*Phragmites sp.*) culms. (**a**–**c**) are modern *P. australis* from Milan Castle (Site 8), and (**d**–**f**) are *Phragmites* from the Tang Dynasty Sunji beacon tower (Site 12). (**a**) and (**d**) are external views showing typical culm epidermal features with two distinct cell types—the elongated rectangular cells and the short silica cells (indicated by black arrows with white margins). The modern sample is covered with epicuticular wax crystals (white arrow with black margin), whereas the ancient sample only has some wax remaining. Scale bar = 50 μm. (**b**) and (**e**) are internal views. The inner membranes are indicated by stars. Black arrows with white margins indicate vessel elements. Ancient sample shows certain degree of decay as indicated by cracked membranes and unclear cell boundaries. Scale bar = 500 μm. (**c**) and (**f**) are also internal views, showing vessel elements with lignified spiral secondary walls and surrounding parenchyma cells. Except for certain degree of degradation in the ancient sample, the shape and size of both vessel elements and parenchyma cells are almost identical. Scale bar = 100 μm.

In the vast arid regions of central Asia, human livelihood is ultimately dependent on oases that supply freshwater to sustain both natural vegetation and agricultural crops^17^. Ancient walls, small and large forts, beacon towers, lookout platforms, and other structures were constructed and fortified using locally available plants harvested from such oases^5^, with *Phragmites* Adans. (Poaceae family), the cosmopolitan common reed, being the most conventional genus used in fascines as natural building material (Fig. 3). *Phragmites* is a highly successful C_3_ plant genus that has considerable variation with high phenotypic plasticity, a wide geographic distribution, and the ability to occupy aquatic and marginal habitats under various climate conditions^18,19^. When available, *Phragmites* was mixed with hardwood species in Great Wall fascines for added strength and durability^1^. Despite biochemical analyses on tissues from the species *Phragmites australis* (Cav.) Trin. ex Steud.^20–22^, reports on pollen^23^ and phytoliths^24^ of *Phragmites* in archaeological contexts, and the identification of a rope made from culms (stems) of *Phragmites* at the Gumugou Cemetery, a site dated to 3800 years cal BP and ~ 70 km east of Lop Nur^25^, no molecular characterization or isotope measurements have been taken on the ancient remains of *Phragmites* collected directly from the Great Wall itself.

Today, a large portion of northwestern China, including the Tarim Basin, the Hexi Corridor in Gansu, and the area west of the Helan Mountains of Inner Mongolia, has a semi-arid to arid continental climate with hot summers and cool, dry winters characterized by low rainfall and prolonged droughts^26,27^ (Fig. S1). This region, and specifically the eastern Tarim Basin, is a key geographical crossroad between Central and East Asia, holding political, military, cultural, and economic significance historically^6^. It has also been subject to both imperial expansion and agricultural intensification over the last two millennia^15,28–31^. Key to this expansion was the distribution of oases, unique landscapes that integrated natural and human-engineered ecosystems, which sustained human populations, economic growth, and political agendas^17^. Desertification brought on by natural climate changes and amplified by human activities^32–34^ intensified evapotranspiration^35^ and the proliferation of desert and xeric shrub-land habitats in the region^36^, reducing available oases today. Such changes have potential historical corollaries, however, as exhaustive irrigation farming and an overdrawing of highland tributaries during the Han Dynasty were believed to have changed local hydrology and caused salinification of lakes bordering the Tarim Basin^30,37^.

Extensive lacustrine^31,38–45^, speleothem^46,47^, and ice core^48,49^ records from northwestern China demonstrate long-term hydroclimate and environmental changes since the Han period. Yet, these records are distal to archaeological sites and do not necessarily reveal on-site (i.e., proximal) ecological subtleties that would have existed at various locations along oases in otherwise arid northwestern China when ancient Great Wall segments were built. In fact, there are no on-site paleoenvironmental reconstructions from northwestern China that speak directly to the creation and maintenance of altered ecosystems by the Han or later dynasties.

Here, we characterize the molecular preservation and distribution of plant wax *n*-alkanes and measure bulk carbon (δ^13^C) and nitrogen (δ^15^N) isotope ratios of accelerator mass spectrometry (AMS) dated *Phragmites* sampled directly from 13 Great Wall sections or beacon towers constructed during the Han, Jin, Tang, or Song Dynasties in today’s Gansu and Xinjiang. Our on-site data provide novel evidence for the source and diversity of reeds used in Great Wall building activities. We compare the ancient data with biochemical information obtained from modern *P. australis* available from the study area to reveal climatic and environmental dynamics during early Chinese historical periods with a focus on the rate, timing, and cause of hydrological changes. Biomolecular analyses of these organic materials preserved within ramparts may provide direct evidence of climatic and environmental conditions at specific historical points along the Great Wall. This study also highlights the future potential of these in situ material as valuable biogeochemical archives for studying human-altered ecosystems and hydrology.

## Results

### Accelerator mass spectrometry (AMS) ^14^C dating

To constrain the chronology of each sampling location, we obtained eight AMS radiocarbon ages from four sites (Table 1 and see “Methods”). We determined that the Majuanwan Great Wall (Site 7 on Fig. 1), and thus the eastern cluster sites in Gansu (Sites 1–7), were built during the Han Dynasty between 132 and 116 BC (2082–2066 cal. B.P., 95% prob.), adhering to Han era historical records and artifacts associated with these structures (Supplementary Materials). The Yakelun and Sunji beacon towers (Sites 11 and 12) in Xinjiang were constructed during the Tang Dynasty and date between 677 and 726 AD, confirming recent archaeological findings^50,51^. Finally, and somewhat surprisingly, the Sishilidadun Beacon Tower (Site 14) dates to the Song Dynasty, 1030–1160 AD, when the eastern Tarim Basin was governed by the Western Liao Dynasty.

**Table 1:** AMS ^14^C dates obtained on ancient reeds from select study sites.

Lab code	Sample code	Site number	^14^C age (BP)	Calibrated range (2σ)	Median date	Dynasty
XA53291	XJP16-24	7	2082	337–53 BC	132 BC	Han
XA53292	XJP16-28	7	2066	174–49 BC	116 BC	Han
XA53293	XJP16-46	11	1224	667–774 AD	726 AD	Tang
XA53294	XJP16-47	11	1253	657–773 AD	697 AD	Tang
XA53295	XJP16-48	12	1266	655–773 AD	684 AD	Tang
XA53296	XJP16-49	12	1273	653–773 AD	677 AD	Tang
XA53297	XJP16-53	14	920	994–1149 AD	1030 AD	Song
XA53298	XJP16-54	14	790	1047–1217 AD	1160 AD	Song

Other sampling locations were previously dated. The Milan Castle Heritage Site (Site 8) was constructed during the Tang Dynasty and radiometrically dated to ~ 770 AD^28^. The Yingpan City Buddha Tower and Burial Site (Site 9) was radiocarbon dated to a median age probability of 305 AD in the Jin Dynasty^52^, consistent with the funerary artifacts found in the Yingpan cemetery^53^. Due to similar archaeological contents found in burials^54,55^, the nearby Yingpan City Heritage Site (Site 10) is assigned to the Jin Dynasty as well, although it is likely that Yingpan City was continuously occupied since the Han Dynasty. Finally, we attribute the Tahaqi Beacon Tower (Site 13), to the Song Dynasty period due to its proximity and similar style of construction to Sishilidadun.

### Pyrolysis gas chromatography mass spectrometry (Py-GC-MS)

**Figure 4:**
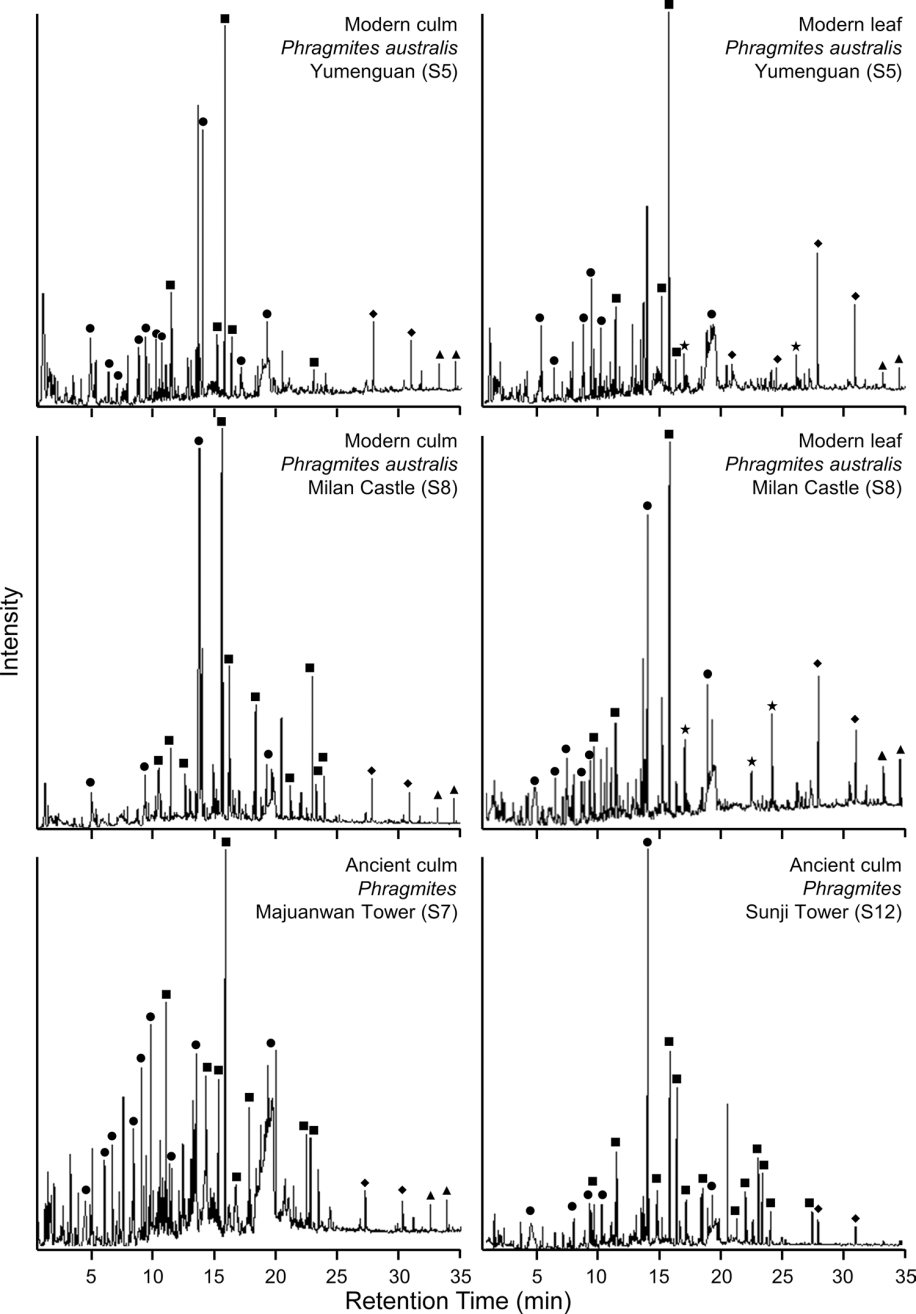
Partial ion chromatograms. The Py-GC-MS analysis of modern *Phragmites australis* culms and leaves and ancient *Phragmites* culms showing the distribution of (black circle) Polysaccharide, (black square) lignin, (black rhombus) fatty acid, (black star) amino acid, and (black triangle) *n*-alkanes. The “S,” as in S7, stands for “Site.” See Data S2 for compound identifications.

With minor variations in pyrolysis moieties among different sites, ancient *Phragmites* culms (*n* = 6) consistently exhibit excellent molecular preservation with abundant labile biomolecules (Fig. 4 and Fig. S2; Data S2). Lignin and polysaccharide pyrolysates dominate the molecular composition of culms from Han and Tang Great Wall segments, beacon towers, and fortifications and have a similar distribution pattern as modern *P. australis* (*n* = 4, Fig. 4). Ancient samples do, however, contain compounds that are not identified in modern analogs, such as apocynin and desaspidinol, though except for one sample, they lack amino acids (Data S2). Apocynin and desaspidinol likely derive from lignin decomposition products^56^ or as possible indicators of hardwood cross-contamination^57^, as hardwood species such as *Tamarix* sp. were sometimes mixed with *Phragmites* in Great Wall fascines^1^.

Major pyrolysate compounds identified in all samples are benzene and furan derivatives, phenol derivatives, and indole derivatives of amino acids (Data S2). Lignin moieties contain phenol, methyl and methoxy phenol, vinyl phenol, and vanillin, while polysaccharide moieties include furans and furfural, benzofuran, and levoglucosan. Lipids are detected primarily as *n*-alkanes and palmitic (C_16_) and stearic (C_18_) acids, but dodecanoic (C_12_) and tetradecanoic (C_13_) acids were identified in one modern *P. australis* leaf collected near Yumenguan (Site 5). Indoles (e.g., Indole, 3-methyl-) indicate the presence of amino acids in modern *P. australis* samples. There are some variations in compound distribution among ancient samples collected from different sites (Figs. 4 and S2). For example, ancient culms from Han Dynasty Yumenguan (Site 5), have fewer lignin derivatives but contain identifiable fatty acids compared to contemporary Majuanwan (Site 7) samples which have more abundant lignin derivates but fewer overall polysaccharide compounds (Data S2). Nevertheless, the Tang era Sunji Tower sample has a similar suite of pyrolysis products as the older Yumenguan and Majuanwan culms, further highlighting the excellent preservation of Han era samples.

Even though site-specific pyrolysates may vary, the high polysaccharide and lignin fiber content of ancient *Phragmites*, compounds that provide strength and durability to culms, coupled with the arid regional climate^15^, resulted in the long-term preservation of these plant parts in the wall fascines as revealed with SEM (Fig. 3). This excellent preservation also suggests that the absence of leaves and inflorescences/infructescences in the walls was an intentional sorting process for high fiber material, as these plant parts would have also been preserved had they been used in wall construction.

### Lipid concentration and distribution

Culms from ancient wall segments or beacon towers have wider *n*-alkane distributions than their modern *P. australis* analogs even though they contain relatively lower quantities of lipids. The concentration of *n*-alkanes (C_21_–C_33_) in ancient culms is approximately 12-times lower than that of modern samples (Data S1), having between 12 and 8610 μg (1160 ± 1702 μg/g; *n* = 38) per gram of dry material (μg/g) compared to the 4163 to 32,296 μg/g (14,065 ± 8042 μg/g; *n* = 12) measured in modern plants. Overall, there is a significant difference in C_21_–C_33_ *n*-alkane abundance between modern and ancient samples as shown by a Student’s t-test (two-tailed, *p* = 0.0002) and Mann–Whitney *U* test (*p* = < 0.0001).

**Figure 5:**
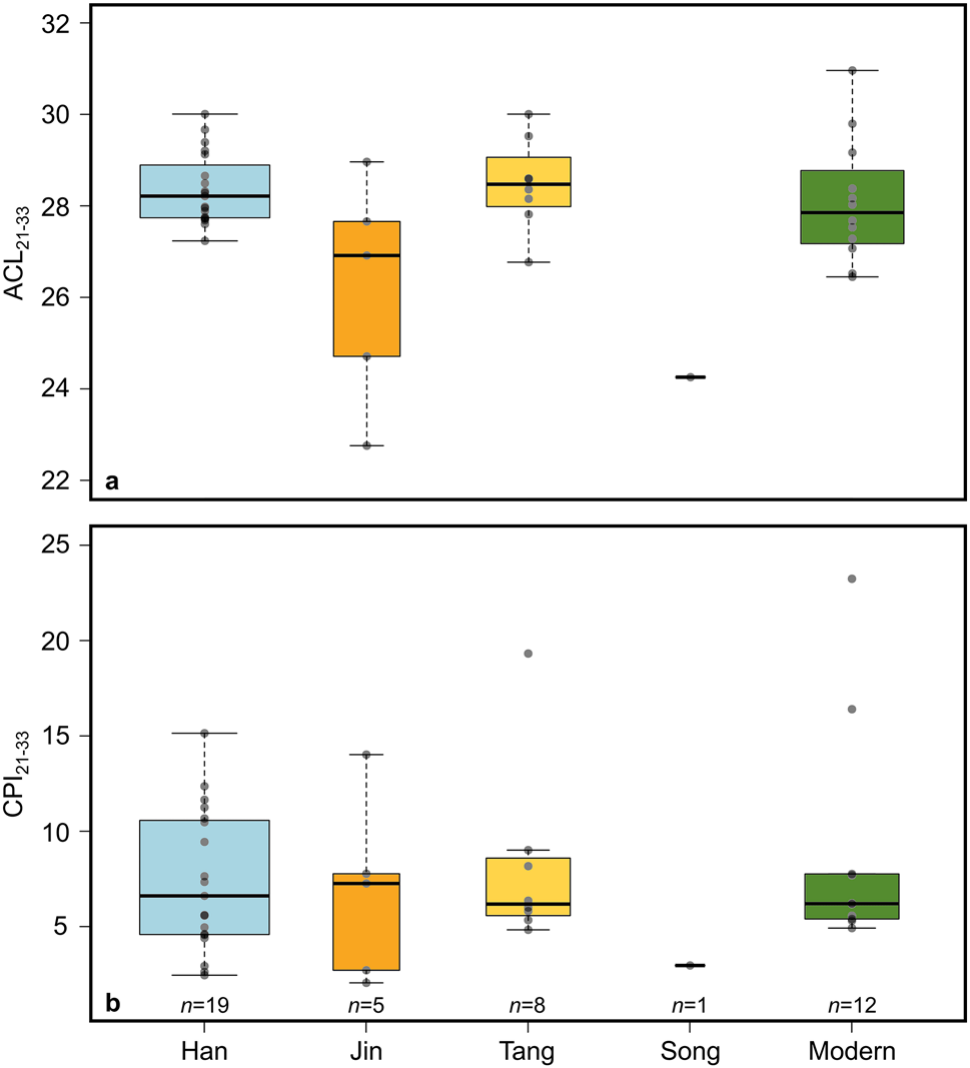
Box plots by age for ACL and CPI. (**a**) Average chain length of the C_21_-C_33_ *n*-alkanes (ACL_21-33_). (**b**) Carbon preference index of the C_21_-C_33_ *n*-alkanes (CPI_21-33_). The thick black lines show the mean values from each period, while the widths of the boxes are proportional to the square-roots of the number of observations.

The lower concentration of *n*-alkanes in Great Wall samples is expected given their antiquity. However, the carbon preference index of the C_21_–C_33_ *n*-alkanes (CPI_C21–C33_), a metric used to examine the odd-over-even carbon number predominance and as an indicator for hydrocarbon maturity^58^, indicates that no significant degradation occurred among the longer chain *n*-alkanes from ancient samples. Both modern and ancient CPI_C21–C33_ values are ≥ 2.0 (Fig. 5, Data S1), which is typical of plant-derived CPI values^59,60^. There is also no significant difference between modern and ancient reed CPI in a Student’s t-test (two-tailed, *p* = 0.634) and Mann–Whitney *U* test (*p* = 0.827). Like lignin and polysaccharide, lipid preservation is contingent on the dry regional climate which likely facilitated the preservation of these organic archaeological remains over time^15,61,62^.

**Figure 6:**
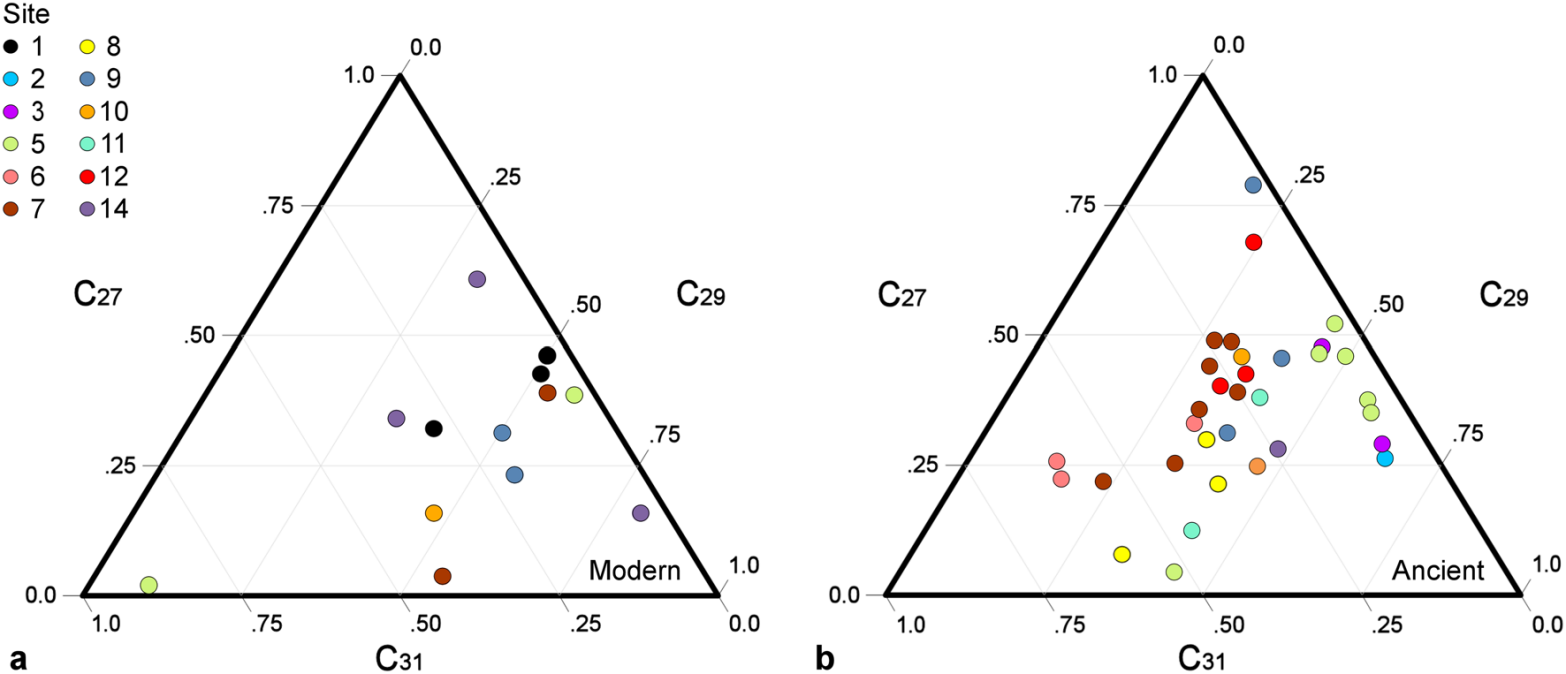
Ternary diagrams of the C_27_, C_29_, and C_31_ *n*-alkanes. (**a**) Relative abundances of each *n*-alkane compound for **a.** modern *P. australis* and **b.** ancient Great Wall *Phragmites***.** Site number corresponds to locations and color scheme in Fig. 1.

Figure 6 shows the ternary diagrams of the C_27_, C_29_, and C_31_ relative abundances for *n*-alkanes from ancient *Phragmites* and modern *P. australis*. The wider chain-length distribution of *n*-alkanes in ancient reeds contrast with those of modern samples, suggesting that ancient *Phragmites* used in the construction of the Great Wall were harvested from habitats that were likely more diverse and growing under cooler and wetter climate conditions relative to today, as chain-length distribution in *P. australis* has been shown to be a function of temperature and water availability^22,63^. Of the 33 ancient samples containing enough lipid material for GC-FID analysis, 13 (39.4%) have C_27_ as the most dominant *n*-alkane, while C_29_ and C_31_ are most abundant in 9 (27.2%) and 8 (24.2%) samples, respectively. The sample from Sishilidadun has C_21_ as the most dominant compound. This wider *n*-alkane distribution contrasts with modern reeds where 9 of the 12 samples (75%) have the C_29_ homologue as the most dominant compound, while only 2 (16%) samples from Sishilidadun have C_27_ as the most abundant alkane, and 1 sample (8%) from Yumenguan has C_31_ (Fig. S3).

The average chain lengths (ACL_21–33_) for Han and Tang aged reeds overlap with the distribution of modern ACL_21–33_ values (Fig. 5) and are consistent with previous reports of modern *P. australis* ACL from China^20–22,64^. ACL_21–33_ values from the Jin and Song Dynasty samples, however, are progressively lower than their modern counterparts (Fig. 5). Although there was no significant difference in ACL_21–33_ values between all modern and ancient reeds in a Student’s t-test (two-tailed, *p* = 0.617) and Mann–Whitney *U* test (*p* = 0.616), ACL_21–33_ tracks higher in all modern samples from individual sites where both were sampled, except Majuanwan (Fig. S4). ACL values have been shown to correlate with higher growing season temperature and aridity^65–67^, and therefore, the higher ACL values in modern plants are consistent with the nearly 1 °C increase in regional temperature over the past 50 years^68^ and the enhanced aridity that has resulted from intensive irrigation farming that began in the middle of the 20th Century in northwestern China^37^. Selective pressures may favor the production of longer *n*-alkane chain lengths under hot or arid conditions^69,70^, and the extant *P. australis* likely suffer from water stress brought on by significant evapotranspiration and elevated 21st Century temperatures, both of which drive ACL values higher. The uncertainty associated with using plant wax distribution, CPI, and ACL as environmental indicators, however, necessitates the application of stable isotope measurements to make stronger inferences on climatic and environmental change.

### Bulk carbon isotope analysis

**Figure 7:**
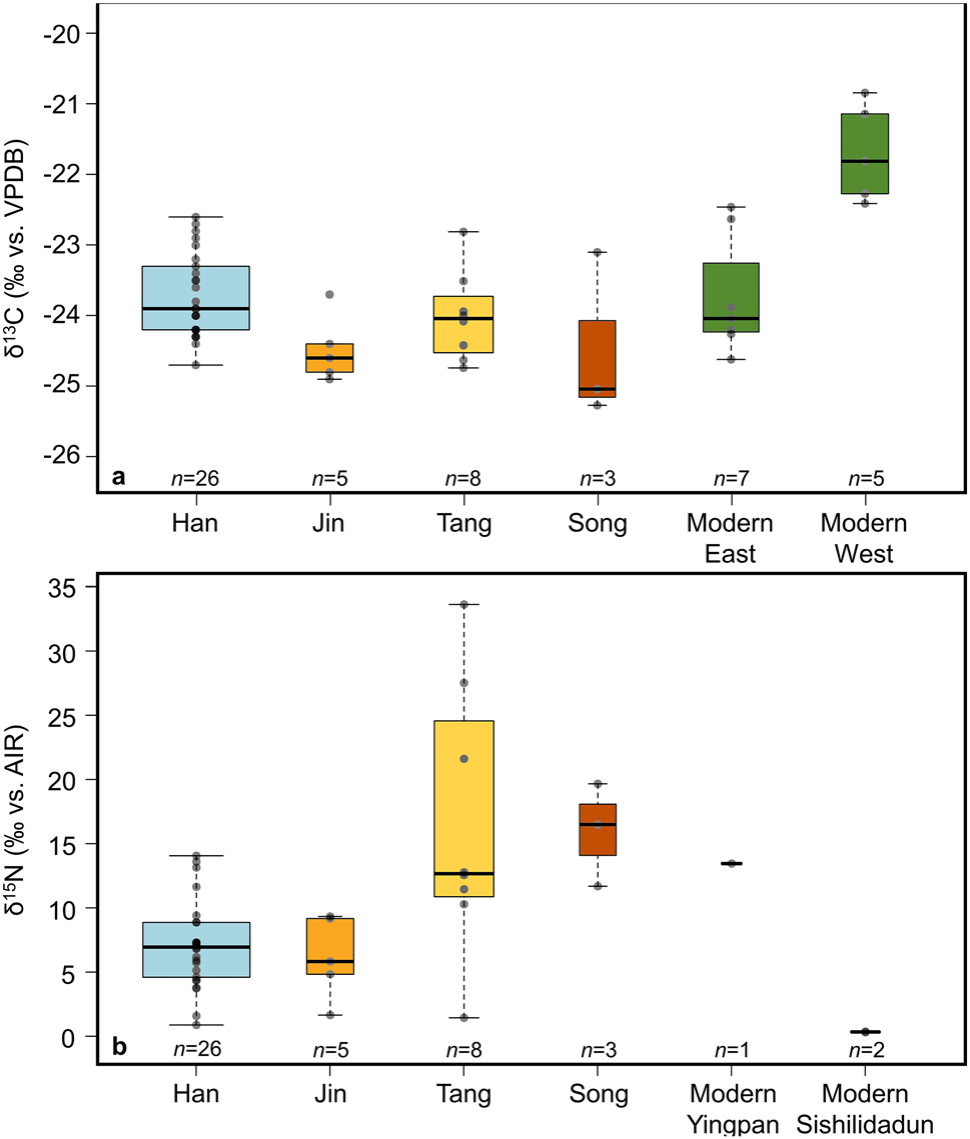
Bulk isotope data box plots by age. (**a**) Han (Sites 1–7), Jin (Sites 9 and 10), Tang (Sites 11 and 12), and Song (Site 14) δ^13^C data from Great Wall *Phragmites*. Modern data includes all extant *P. australis* separated into eastern and western clusters. (**b**) Han, Jin, Tang, and Song δ^15^N data from Great Wall *Phragmites* and modern *P. australis* from Sites 10 and 14. The thick black lines show the mean values from each period, while the widths of the boxes are proportional to the square-roots of the number of observations.

Ancient reeds from Han (Sites 1–7), Jin (Sites 9 and 10), Tang (Sites 8, 11, 12), and Song (Site 14) samples yield bulk δ^13^C values between − 25.3‰ and − 22.6‰ (− 23.9 ± 0.7‰; *n* = 42), while modern bulk δ^13^C values, corrected for the Suess effect, range between − 24.6 and − 20.8‰ (− 22.9 ± 1.3‰; *n* = 12) (Fig. 7; Data S1).

**Figure 8:**
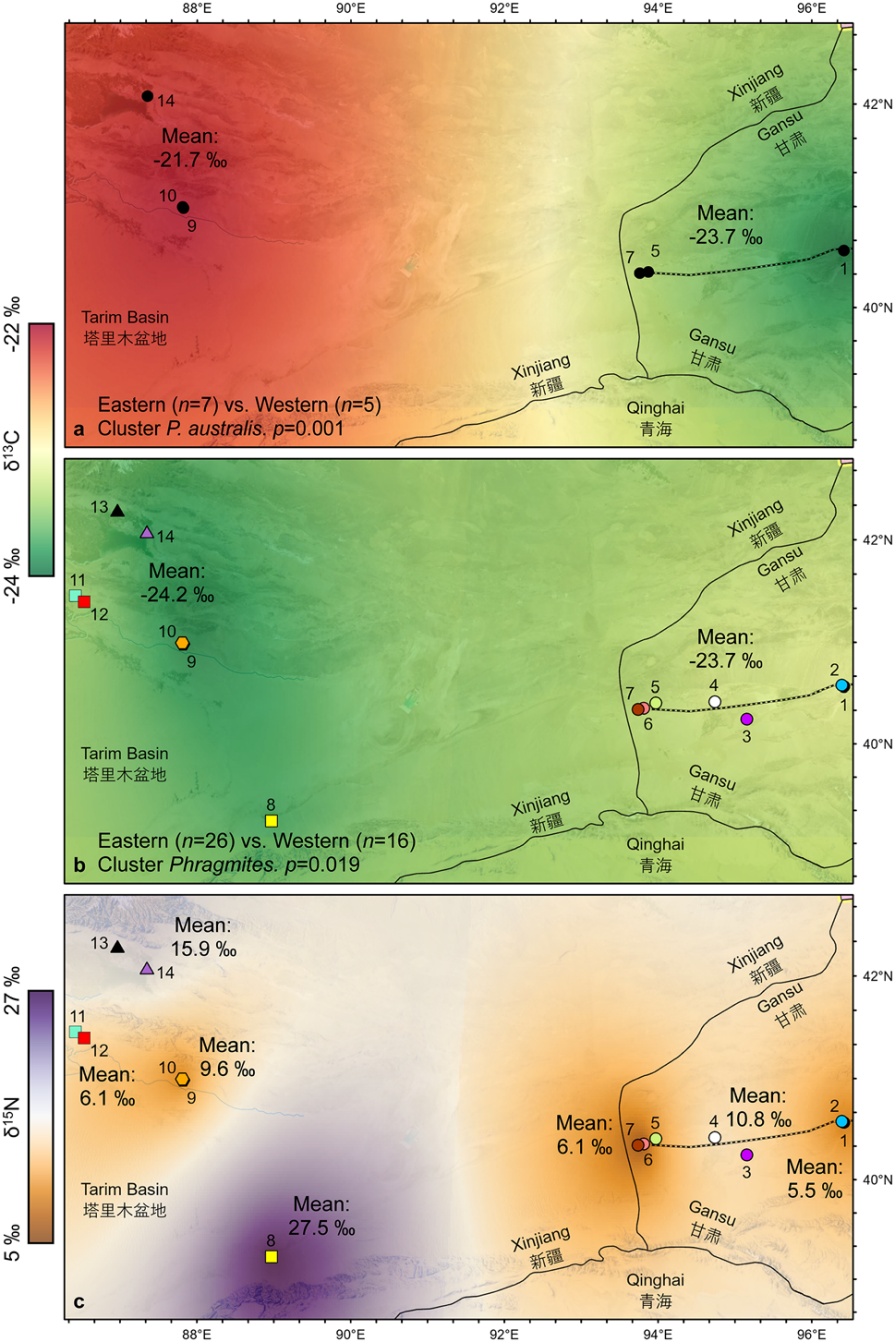
Carbon and nitrogen isoscapes. (**a**) Modern *P. australis* showing the sharp, 2.0‰ contrast in mean δ^13^C between eastern and western clusters and the two-tailed Student’s t-test *p*-value from Table 2. (**b**) Ancient Great Wall *Phragmites* have more homogenous δ^13^C values across eastern and western clusters, with western samples being 0.5‰ lighter than their eastern counterparts on average. Also shown is the two-tailed Student’s t-test *p*-value for all ancient *Phragmites* from eastern (Sites 1–7) and western (Sites 8–14) clusters. Although both *p*-values are < 0.05, (**a**) and (**b**) demonstrate the large 2.5 ‰ difference between modern and ancient samples from the western cluster following 21st Century warming. (**c**) Highly variable δ^15^N values likely suggest evidence of fertilization in some sites, specifically Milan Castle (Site 8) and Sishilidadun Tower (Site 14). Maps were created using the Kriging feature in ArcGIS Pro, whereby color variation in the surface reflects the spatial correlation between δ^13^C or δ^15^N values.

There is a clear geographic patterning in δ^13^C signatures of modern *P. australis* samples. Modern samples exhibit significant differences in δ^13^C values between samples from eastern (*n* = 7) and western (*n* = 5) sites (Student’s t-test two-tailed, *p* = 0.001; Mann–Whitney *U* test, *p* = 0.003; see Table 2), as western samples are 2.0‰ higher on average than eastern *P. australis*, having mean corrected δ^13^C of − 21.7‰ and − 23.7‰, respectively (Figs. 7, 8a). An opposing pattern is observed in ancient *Phragmites* samples, however, as the mean Han Dynasty δ^13^C value from the east (*n* = 26) is 0.5‰ heavier than western (i.e., Jin, Tang, and Song; *n* = 16) samples (Figs. 7, 8b; Table 2). Interestingly, there is no significant difference between corrected modern and Han δ^13^C values in the eastern cluster (Table 2), as both have means of − 23.7‰. On the other hand, corrected modern samples from Xinjiang are 2.5‰ higher than their ancient analogs with modern *P. australis* having a mean δ^13^C value of − 21.7‰ compared to the Jin, Tang, and Song Dynasty sample mean δ^13^C values of − 24.2‰ (Table 2).

**Table 2:** Student’s t-test (top/right) and Mann–Whitney *U* test (bottom/left) *p*-value results for bulk δ^13^C from ancient and modern *Phragmites*.

δ^13^C*	Han (*n* = 26)	Jin (*n* = 5)	Tang (*n* = 8)	Song (*n* = 3)	Mod. East (*n* = 7)	Mod. West (*n* = 5)	Mod. all (*n* = 12)
Han (− 23.7‰)	–	0.016	0.273	0.398	1.000	0.002	0.048
Jin (− 24.5‰)	0.011	–	0.164	0.990	0.075	< 0.001	0.002
Tang (− 24.0‰)	0.207	0.222	–	0.595	0.472	< 0.001	0.018
Song (− 24.5‰)	0.209	0.571	0.376	–	0.399	0.035	0.135
Mod. East (− 23.7‰)	0.877	0.106	0.536	0.267	–	0.001	n/a
Mod. West (− 21.7‰)	0.001	0.008	0.002	0.036	0.003	–	n/a
Mod. all (− 22.9‰)	0.057	0.014	0.047	0.070	n/a	n/a	–

*Mean reported as ‰ vs. VPDB.

This 2.5‰ heavier δ^13^C value of modern *P. australis* in the western cluster and the uniformity in eastern modern and Han aged samples, suggest a differential rate of environmental change on opposite sides of Lop Nur. We attribute the higher δ^13^C values of western *P. australis* to ^13^C-enrichment that occurrs in plants growing in environments with higher temperatures and rates of evapotranspiration^71,72^. Plants in arid or hot environments are proportionally enriched in ^13^C compared to those growing in cooler or well-watered locations because the rate of water loss intensifies as plants must augment stomatal conductance to preserve leaf water^71,72^. Annual mean temperature and precipitation at Yuli City, which represents our modern western samples’ climate parameters, averages 12.1 °C and 37.2 mm, respectively. This is ~ 5 °C warmer and half the annual precipitation of that in Yumen City from east of Lop Nur (Fig. S1). Thus, the extensive aridity and higher evapotranspiration rate in Xinjiang has a significant fractionation effect on bulk carbon isotope values, resulting in the mean 2.5‰ difference between modern and ancient samples, as well as the mean 2.0‰ difference between modern samples from Xinjiang and Gansu. It is reasonable to infer that the rate of change over time has increased as bulk δ^13^C from modern *P. australis* collected at Yingpan City (Sites 9 and 10) is 2.7‰ heavier on average than Jin Dynasty Great Wall culms from the site, while modern samples from the Sishilidadun Beacon Tower of the Song Dynasty (Site 14) are on average 2.8‰ higher than ancient reeds from this location.

### Bulk nitrogen isotope analysis

Ancient reed samples yield large variations in bulk δ^15^N, with values ranging from 0.8 to 33.5‰ (9.3 ± 6.7‰; *n* = 42). Extremely heavy δ^15^N values were obtained in samples from the Tang Dynasty Milan Castle Heritage Site (Site 8, 27.5 ± 6.0‰; *n* = 3) and in the Song Sishilidadun Tower (Site 14, 15.9 ± 4.0‰; *n* = 3). In general, δ^15^N values in Han (6.9 ± 3.3‰; *n* = 26) and Jin (6.1 ± 3.2‰; *n* = 5) era samples are significantly different from Tang (16.3 ± 10.4‰; *n* = 8) and Song (15.9 ± 4.0‰; *n* = 3) aged samples (Table 3), due to the heavy δ^15^N values obtained from Milan Castle and Sishilidadun (Data S1). This may suggest that the δ^15^N composition of plant or soil nitrogen in Tang aged samples was influenced by temperature and precipitation at the large, regional scale^73–75^, but also by human activities at the small, site-specific scale. The extremely high δ^15^N values at Milan Castle and Sishilidadun Tower coincide with other evidence of high (i.e., ≥ 15‰) δ^15^N values from Xinjiang^52,76^, indicating that human activities such as crop fertilizing, in addition to climatic parameters, may have elevated δ^15^N values at these locations.

**Table 3:** Student’s t-test (top/right) and Mann–Whitney *U* test (bottom/left) *p*-value results for bulk δ^15^N from ancient and modern *Phragmites*.

δ^15^N^*^	Han (*n* = 26)	Jin (*n* = 5)	Tang (*n* = 8)	Song (*n* = 3)	Mod. East (*n* = 0)	Mod. West (*n* = 3)	Mod. all (*n* = 3)
Han (6.9‰)	–	0.603	0.041	0.066	n/a	0.652	n/a
Jin (6.1‰)	0.809	–	0.029	0.023	n/a	0.782	n/a
Tang (16.3‰)	0.009	0.030	–	0.918	n/a	0.096	n/a
Song (15.9‰)	0.011	0.036	0.921	–	n/a	0.108	n/a
Mod. East (n/a)	n/a	n/a	n/a	n/a	–	n/a	n/a
Mod. West (4.6‰)	0.299	0.571	0.194	0.200	n/a	–	n/a
Mod. All (n/a)	n/a	n/a	n/a	n/a	n/a	n/a	–

*Mean reported as ‰ vs. AIR.

The culms used for the construction of the Milan Castle yield an exceptionally high mean δ^15^N value of 27.5 ± 6.0‰, which is about 12‰ heavier than that of Sishilidadun. The 15.9 ± 4.0‰ δ^15^N from ancient Sishilidadun stands in exceptional contrast to the modern 0.24 ± 0.04‰ value obtained from extant *P. australis* growing at the site. The only other modern δ^15^N value obtained, 13.4‰, comes from the Yingpan City Heritage Site (Site 10). As for other ancient samples, the Cang Ting Sui Beacon Tower (Site 5) and wall segments at the Great Wall Heritage Site (Site 6), locations which are only 5 km apart, have mean δ^15^N values of 8.3‰ and 2.0‰, respectively, showing the large variability and local variations between sites (Fig. 8c). Based on mean δ^15^N of all ancient *Phragmites* except those from Milan Castle and Sishilidadun (7.2 ± 3.6‰; *n* = 36), the local differences in δ^15^N more likely reflect the use of fertilizers around large population centers in the past, which would have resulted in potential agricultural runoff that drove δ^15^N values higher in wild reeds that were then harvested for wall building. Fertilizers derived from manures or guano are enriched in ^15^N and have a significant impact on plant nitrogen isotopic values^73,77–79^.

## Discussion

Although the rammed-earth and reed fascine segments of the ancient Great Wall do not elicit the amount of visual attraction as the brick and stone masonry of the Ming Dynasty, they offer a wealth of unique scientific information on the sourcing of natural organic building materials in addition to paleoclimatic and environmental signals. Our AMS results (Table 1) confirm unsynchronized ages for construction of Great Wall segments along the Shule River in Gansu Province and the beacon towers and associated fortifications along the Kongque River in Xinjiang. As our radiometric ages corroborate with archaeological findings^11–14^ (Supplementary Materials), we are confident that the ancient reeds analyzed here were in fact original Great Wall building material.

The different ages obtained from the Xinjiang beacon towers are somewhat surprising. On the one hand, the Tang aged material from Sites 11 and 12 confirm recent archaeological findings from a nearby tower^50,51^, whereas the younger age obtained from Sishilidadun (Site 14), one of the northern-most beacon towers along the Kongque River, suggests that wall-building activities lasted into the post-Tang era and may have been a defensive practice adopted by nearby states like the Qocho Uyghur Kingdom or imported from northeastern China when the Western Liao Dynasty was established in Central Asia. Nevertheless, our new radiometric dates shed light onto the previously conflicting archaeological age uncertainties for these ancient structures.

Both the pyrolysis and *n*-alkane data demonstrate the exceptional preservation of the ancient reeds used in Great Wall construction, despite the lower quantity of certain biomolecules in archaeological samples. Desiccation enhances preservation for plant material at both structural and molecular levels^61,62^, so it is expected that the dry climate of northwestern China helped facilitate the longevity of ancient reeds protected within wall ramparts. The excellent molecular preservation of these ancient reeds is confirmed with SEM observations (Fig. 3). Both external and internal views of ancient culm samples show identical cellular features, including epicuticular wax crystals, epidermis, internal membranes, vessel elements, parenchyma cells, fibers, etc., with their modern counterparts having only slight degradation. The preservation of these in situ organic material, therefore illustrates the potential of using these site-specific, common reeds as a proxy source for studying climatic and environmental change at a smaller, local scale. As fragments of common reeds are included in the construction mix of building materials in ancient, arid Central Asia, they will be essential to archaeological studies seeking to illuminate the influence of human activities on the ecology and distribution of oases in the eastern Tarim Basin since the Han period. Additionally, this technique can be adopted elsewhere and applied to any ancient ruin that preserves organic materials.

While northwestern China has become warmer and dryer since the Han Dynasty^30,48,80^, the large δ^13^C offset between modern *P. australis* and Jin, Tang, and Song Dynasty aged *Phragmites* indicates variability in the localized ecological response to changing climate and surface-water hydrology. We suggest that the uniformity in ancient δ^13^C values recorded in Great Wall *Phragmites* culms from across the eastern Tarim Basin (Fig. 8a) may reflect the once wider availability of regional oases, likely due to previous homogeneous wetter climatic conditions brought on by a stronger Asian monsoon that penetrated further into western China^32,33,44,48^. On the other hand, the uniformity in bulk δ^13^C between Han aged and modern *P. australis* samples collected in Gansu suggests that surface-water hydrology within the Shule River catchment has been relatively consistent over the last two millennia. This contrasts with the high values of bulk δ^13^C in modern samples collected from the western cluster that now suffers a greater degree of environmental stress due to elevated temperatures and a higher degree of aridity and evapotranspiration, suggesting that climatic conditions in Xinjiang have drastically changed over the past century.

The regional environmental change in China’s northwestern frontier is of explicit concern in the discussion of various episodes of migrations^81–83^ and cross-cultural exchanges of technologies, military, farming, and pastoral activities, with the eastern Tarim Basin acting as a crossroad in those narratives^84,85^. Both Han and Tang Dynasties were periods of significant expansion of the Chinese empire into the northwest, a phenomenon that historians largely attribute to a unified central power, economic growth, cultural achievements, and more importantly, strong military capabilities^86^. However, recent studies have pointed out the possible link between climate change and state affairs and regional conflicts, especially along China’s northwestern border between nomadic and farmer groups^87^. Our data provide additional evidence linking the availability of locally sourced material with wall building activities during the Han, Jin, and Tang periods, with conflict possibly incentivising the construction of walls or beacon towers^87^. Nevertheless, further research is needed to determine whether this only occurred when climate conditions sustained sizeable oases in the region, or other ways in which climate played a role in shaping historical change and development^88–90^.

The Tarim Basin’s Taklamakan Desert is characterized by extreme aridity and extensive evapotranspiration, which results in large plant ^15^N enrichment due to intensive evaporation and low mean annual precipitation^91^. Located at the center of the Eurasian continent, the Taklamakan Desert is the world's second largest shifting sand desert, with evaporation reaching as high as 1500 mm yearly and annual precipitation being only between 50 and 80 mm on the basin’s edges and 17–25 mm at the center^68,92^. As δ^15^N values of plant roots, plant litter, and soil organic matter increase with decreasing precipitation^73,93^, the entire Tarim Basin has some of the most elevated terrestrial δ^15^N values in Eurasia^52,76,91^, as exemplified by the δ^15^N value of 13.4‰ from modern *P. australis* sampled at Site 10.

The extremely high δ^15^N values at the Tang-era Milan Castle, however, suggest that fertilizers derived from manure or guano had a significant effect on wild plant nitrogen isotopic values^73,77–79^. The mean 27.5 ± 6.0‰ obtained from Milan Castle could only be reached through anthropogenic input, whether deliberately or as agricultural runoff. The high, 15–20‰ δ^15^N values of Yingpan Man and associated wheat/barley, broomcorn millet, grape, goats, and sheep^52^, all suggest that high nitrogen values correspond to human subsistence strategies, specifically when compared to our relatively low results from Yingpan City (Sites 9 and 10, 6.1 ± 3.2; *n* = 5), and from the site’s modern *P. australis* δ^15^N value of 13.4‰. The δ^15^N data from Yingpan City would therefore suggest that local *Phragmites* were not purposely fertilized here, nor did they grow in an area influenced by agricultural runoff. Though we are currently unable to determine whether *Phragmites* used in the construction of Milan Castle were purposely fertilized and deliberately managed for wall construction, or had naturally collected nutrients from agricultural runoff, the extremely high value here compared to sites like Yingpan City suggests that the possible manuring hypothesis deserves further investigation.

It has been suggested that widespread irrigation projects and agricultural intensification quickly decreased the amount of surface water along the eastern Tarim Basin, reducing overall lake levels^44,80^. However, the uniformity in our ancient *Phragmites* δ^13^C values indicates that surface-water availability, and the extent of oases, was more homogenous from the Han through Song Dynasties across both sides of Lop Nur (Fig. 8a), implying little change of surface-water hydrology due to human activities during these periods. Moreover, it may have only been relatively recently^37,68^ that elevated temperatures and aridity had a significant influence on plant δ^13^C in our study region, especially in the western cluster samples (Fig. 8a). Our data, therefore, is consistent with recent temperature records in Xinjiang indicating that the Tarim Basin experienced significant, monotonic warming with an average increase of nearly 1 °C from 1955 to 2000, unevenly distributed across time and space^68^, leading to the intense evaporative stress on modern plants.

Our work demonstrates the potential for paleoenvironmental reconstructions applying molecular and biochemical methodologies to organic materials that are well preserved in ancient Great Wall segments and beacon towers in the western frontier from important periods in Chinese history. Our study represents the first attempt to reconstruct the source and local habitats of Great Wall building materials that contain information on the impact of climate change on local environmental settings. Along with other regional and global climate proxies, this data illuminate site-specific environmental records that speak to localized natural or human-induced environmental changes in northwestern China. Building upon this study, more research based on higher resolution sampling strategies of co-occurring organic materials with other molecular isotope climate proxies from newly-surveyed beacon towers in Xinjiang and Inner Mongolia will certainly yield valuable insights into regional archaeology and environmental changes across time and space. Such methods are not limited to northwestern China, however, and can be applied to any ancient structure that was built, at least partially, using locally-sourced organic materials.

## Methods

### Site locations and sampling

Ancient *Phragmites* culms (*n* = 45), identified only to the genus level, were sampled from exposed fascines of remnant wall segments, beacon towers, or fortification ruins (Fig. 2). Modern culms (*n* = 8) and leaves (*n* = 4) belonging to the species *P. australis* were sampled directly from wild plants growing in the study area (Data S1). All available modern *P. australis* populations in the study area were sampled. Both ancient and modern samples were collected from 14 sites with the permission from local archaeology authorities in Gansu and Xinjiang during joint field expeditions in 2011 and 2016 (Fig. 1, Data S1). Geographically, these sites are grouped as eastern (Sites 1–7) and western clusters (Sites 8–12, 14; Site 13 did not yield data), separated by the now dried Lop Nur Lake basin (Fig. 1b). Climatologically, this region represents one of the driest areas in China with mean annual precipitation of only 66.5 mm at Yumen City (40°16′ N, 97°2′ E) and 37.2 mm at Yuli City (41°21′ N, 86°16′ E), localities representing the climate of the eastern and western side of the Lop Nur basin, respectively. There is also a regional mean annual temperature (MAT) difference, with MAT at Yumen City being 7.5 °C compared to 12.1 °C of Yuli City (Fig. S1). However, different paleoenvironmental proxies suggest wetter climate conditions with higher lake levels and precipitation in northwestern China during the Han and Tang Dynasties^38–40,44,47,48,94–98^. In contrast, lake records demonstrate decreased moisture availability and significant landscape change toward the end of the Han Dynasty and shortly afterwards^31,44,98,99^.

Scanning Electron Microscopy (SEM) was performed on culm pieces of both modern *P. australis* and an ancient *Phragmites* from the Sunji beacon tower (Site 12) that were cut with single-edged blades. The external and internal surfaces of these pieces were then coated with a 15 nm thin film of gold and observed using a JEOL JSM-IT200 Scanning Electron Microscope under low vacuum at 15kv.

We only collected samples from wild *P. australis* from Chinese public land guided by Chinese colleagues with permission granted by their respective research institutes in accordance with relevant guidelines and regulations. This species is not considered at Risk of Extinction and is listed as Least Concern by the IUCN Council, thus, no special permission or license was required for collection^100^. All collected specimens were identified by Dr. Qin Leng and voucher specimens (see sample ID numbers in Data S1) were equally divided between the Institute of Earth Environment at the Chinese Academy of Sciences in Xi’an, Shaanxi and at the Laboratory for Terrestrial Environments in Bryant University, Smithfield, Rhode Island.

### Accelerator mass spectrometry (AMS) ^14^C dating

The chronology of the different sampling locations was established through radiocarbon dating on selected plant samples using accelerator mass spectrometry at the Institutional Center for Shared Technologies and Facilities at the Institute of Earth Environment, Chinese Academy of Sciences in Xi’an, China. Dates were calibrated to cal yr before A.D. 1950 (i.e., cal ^14^C yr B.P.) by IntCal20 using the OxCal v. 4.2.3 software at 95% probability or 2 standard deviations (2σ) (Table 1). For sites where radiocarbon was not applied, the chronology of each location was determined using archaeological artifacts and historical texts^11–14^ (Supplementary Material). All available evidence indicates a Han Dynasty age for sampling sites in the eastern cluster (Sites 1–7). Bamboo slips and wooden tablets recovered from beacon towers at Yumenguan (Site 5, Fig. 1), Majuanwan (Site 7, Figs. 1, 2a,b), and Dunhuang^11,13^ date them from 98 BC to 137 AD^101^. Other archaeological artifacts, such as silk textiles, literature, and the construction style of the beacon towers, further attribute these military structures to the Han Dynasty and as key stops along the ancient Silk Road^13,102^. Two AMS dates obtained from Majuanwan yielded radiometric ages of 132 and 116 BC (± 20 years), confirming its Han Dynasty age (Tables 1, S1).

The ages for sampling sites in Xinjiang (Sites 8–12, 14) are grouped into three categories in terms of their chronology (see Supplementary Materials for detailed description):

1. Site 8 (Milan Castle Heritage Site) is radiometrically dated to ~ 770 AD and was constructed during the Tang Dynasty^28^. Sites 11 and 12, beacon towers north of the Kongque River, are also attributed to the Tang Dynasty based upon archaeological findings^50,51^. Our new radiometric dating of four Great Wall *Phragmites* from Sites 11 and 12 yielded calendar years between 677 and 726 AD, placing them within the Tang Dynasty.
2. The Buddha Tower at the Yingpan City Heritage Site (Site 9) was radiocarbon dated to 305 AD, placing it in the Jin Dynasty^52^. A wall section from the Yingpan City Heritage Site (Site 10) was previously assigned to both the Han and Jin Dynasty based upon burials and associated archaeology^54,55^. We attribute Sites 9 and 10 to the Jin Dynasty as Yingpan was likely built during the Han and continuously occupied through the Jin period.
3. The Sishilidadun Beacon Tower (Site 14) is the youngest of the sites studied, as we obtained two AMS ages with radiometric dates of 1030 and 1160 AD. These dates indicate that the structure was built during the Song Dynasty (960–1279 AD), although Song political borders are not known to have extended into the Tarim Basin.

Most of the ancient reed materials used in construction were culms, as leaves have rarely been recovered from these ancient ruins (Fig. 2b). Modern culms and leaves of native *P. australis* were also sampled at six of the sites that contained reed stands near the ancient ruins to serve as modern comparisons (Sites 1, 5, 7, 9, 10, 14). Morphologically, the culms of ancient reeds are indistinguishable from their modern counterparts (Fig. 3). All samples were kept frozen in the laboratory until analyzed.

### Pyrolysis gas chromatography mass spectrometry (Py-GC-MS)

Modern (*n* = 4) and ancient (*n* = 6) plant samples were analyzed using Py-GC-MS to test for the molecular distributions and preservation of organic compounds at Bryant University, Smithfield, Rhode Island. Samples were pyrolized using a CDS 5250 Pyroprobe by combusting at 610 °C for 20 s to convert macromolecular compounds to GC amenable products. Compound detection and identification were performed using an Agilent 7890A GC System equipped with a Thermo TR-1 capillary column (60 m length, 0.25 mm i.d. and 0.25 μm film) coupled to a 5975C Series Mass Selective Detector (MSD). The GC oven was programmed from 40 °C (5 min hold) to 100 °C at 10 °C/min, then to 300 °C at 6 °C/min (25 min hold). Helium was the carrier gas with a constant flow of 1.1 mL/min. The MS source was operated at 250 °C with 70 eV ionization energy in the electron ionization (EI) mode and the MS Quadrupole mass analyzer was set to 150 °C with a scan rate of *m/z* 50–500. Samples were held at the pyroprobe interface for at least 5 min at 300 °C for additional thermal extraction and to remove volatile impurities before gas chromatography. Compounds were identified by comparing their spectra with those reported in the literature^103,104^. Duplicate analyses of each sample were conducted for analytical consistency.

### Chemical analysis of plant wax lipids

Plant culms and leaves were lyophilized and ground, then extracted with Dichloromethane:Methanol (9:1, v/v) using ultrasonication at 40 °C in three, 30-min cycles at the Institute of Earth Environment, Chinese Academy of Sciences, Xi’an. The total lipid extracts were dried under nitrogen and separated into two fractions through silica gel column chromatography using hexane and methanol, respectively, with *n*-alkanes being eluted in the hexane fraction. Quantification of *n*-alkanes was performed using an Agilent 6890 Series instrument equipped with a split-injector, HP1-ms GC column (60 m length, 0.32 mm i.d. and 0.25 μm film), and a Flame Ionization Detector (FID). Samples were injected in split mode (split ratio 4:1) and the GC oven was programmed from 40 °C (1 min hold) to 150 °C at 10 °C/min, then to 315 °C at 6 °C/min (20 min hold). Helium was the carrier gas with a constant flow of 1.2 mL/min. Sample peak areas were compared with an external standard mixture (C_21_, C_25_–C_33_, odd numbered *n*-alkanes, 50 ng/μL) for compound identification and quantification. Specifically, sample *n*-alkane GC-FID peak areas (PA) were converted to concentrations assuming that the response of each compound is identical to that of the standard mixture (approximately 3–5 PA/ng). A calibration curve was not necessary as the range of *n*-alkanes measured were not outside the standard carbon homologue range.

Generally, long-chains (C_27_–C_35_ *n*-alkanes) are most abundant in terrestrial plants^105,106^, while submerged and floating aquatic macrophytes contain more mid-chain compounds (C_23_–C_25_ *n*-alkanes)^107,108^, and short-chains (C_17_–C_21_ *n*-alkanes) are dominant in algae^109,110^. The average chain length, or the weight-averaged number of carbon homologues of the odd-numbered C_21_–C_33_ *n*-alkanes, is used (cautiously) as both biosynthetic source and climate proxies and was calculated as follows:

$$ACL = \frac{{21\left( {C_{21} } \right) + 23\left( {C_{23} } \right) + \cdots + 33\left( {C_{33} } \right)}}{{C_{21} + C_{23} + \cdots + C_{33} }}$$

where C_*x*_ is the abundance of the chain length with *x* carbons. The carbon preference index (CPI) examines the odd-over-even carbon number predominance of hydrocarbons and distinguishes sedimentary organic matter deriving from terrestrial plants and that from bacterial or petroleum sources^60,111–113^, and as an indicator for hydrocarbon maturity^58^. Alkanes deriving from land plants display carbon chains typically (60.7%) with CPI > 5, but have been shown to range between 1 and 99, with most (81.2%) plants having values > 2^59^. Samples containing petrogenic and marine inputs or mature/degraded samples are characterized by considerably lower CPI values of ≤ 1. CPI was calculated using the abundances of odd and even chain lengths from C_21_ to C_33_ with the following formula:

$$CPI = \frac{{\left( {C_{21} + C_{23} + \cdots + C_{31} } \right) + \left( {C_{23} + C_{25} + \cdots + C_{33} } \right)}}{{2 \times \left( {C_{22} + C_{24} + \cdots + C_{32} } \right)}}$$

### Bulk carbon isotope analysis

Culms from modern and ancient reeds were washed with distilled water and treated with 2 M HCl for 24 h at room temperature to remove any potential carbonates before combustion (4 h, 860 °C) in a vacuum-sealed quartz tube in the presence of Ag foil and CuO. The purified CO_2_ gas was then analyzed for carbon isotopes using a Finnigan MAT251 gas mass spectrometer at the Stable Isotope Biogeochemistry Laboratory at the Institute of Earth Environment, Chinese Academy of Sciences, Xi’an. The national standard GBW04407 (δ^13^C_VPDB_ = − 22.43 ± 0.07‰) was analyzed between every twelve samples. The precision of repeated measurements of the laboratory standard was < 0.1‰. Sample carbon isotope ratios (δ^13^C) are expressed as parts per thousand (‰) relative to the international VPDB standard and defined by the following equation:

$$\delta {}^{13}C = \left( {\left( {\frac{{{}^{13}C}}{{{}^{12}C}}Sample \div \frac{{{}^{13}C}}{{{}^{12}C}}Standard} \right) - 1} \right) \times 1000$$

Since modern and ancient reed δ^13^C values were compared, + 1.9‰ was added to all modern values^114–116^ (Data S1). This is to correct for the ^13^C Suess effect, or the differences in atmospheric δ^13^C_CO2_ between the value averaged for 2011 and 2016 of − 8.4‰^117^, and the pre-industrial δ^13^C_CO2_ value of − 6.5‰^118^.

### Bulk nitrogen isotope analysis

The nitrogen isotope ratios of the dried plant samples were determined at the Stable Isotope Biogeochemistry Laboratory at the Institute of Earth Environment, Chinese Academy of Sciences using a FLASH EA1112 elemental analyzer interfaced with a Delta-Plus continuous-flow isotope ratio mass spectrometer (IRMS). All the δ^15^N values used a KNO_3_ reference material (δ^15^N 6.27‰) and an international isotope reference material (IAEA-N3; δ^15^N 4.70‰) to control the analytical accuracy of the EA-IRMS. Repeated analyses of the laboratory soil standards with confirmed δ^15^N values were performed daily to ensure instrumental accuracy. The standard deviation for repeated sample analyses was better than 0.3‰. The δ^15^N of each sample is expressed as ‰ relative to the international AIR standard and defined by the following equation:

$$\delta {}^{15}N = \left( {\left( {\frac{{{}^{15}N}}{{{}^{14}N}}Sample \div \frac{{{}^{15}N}}{{{}^{14}N}}Standard} \right) - 1} \right) \times 1000$$

Finally, two-tailed Student’s t-tests, assuming unequal variances, and Mann–Whitney *U* tests were used to test the significance in differences between sample sets using an Alpha (α) of 0.05 (see Tables 2, 3). Student’s t-tests and Mann–Whitney *U* tests were run using PAST 4.03 (additional nonparametric statistical tests, Kruskal–Wallis and Spearman’s rank, were also used to test significance in differences between sample sets and are presented in the Supplementary Materials). Mean values are reported along with their standard deviations.

## Supplementary Information

Supplementary Information 1.

Supplementary Information 2.

Supplementary Information 3.

## Data Availability

All data supporting the findings of this study are available within the paper and its supplementary material files.
