# Supplementary material for: Ancient Great Wall building materials reveal environmental changes associated with oases in northwestern China
Source: Sci Rep. 2022 Dec 29;12:22517. doi: 10.1038/s41598-022-27071-4 (PMC9800585; doi:10.1038/s41598-022-27071-4)
Supplement: Supplementary file 1 — Supplementary Information 1. [file 41598_2022_27071_MOESM1_ESM.docx]

Supplementary Materials for

**Ancient Great Wall Building Materials Reveal Environmental Changes Associated with Oases in Northwestern China**

Robert Patalano*, Jing Hu, Qin Leng, Weiguo Liu, Huanye Wang, Patrick Roberts, Michael Storozum, Lin Yang, and Hong Yang*

*Corresponding author. Email: patalano@shh.mpg.de, hyang@bryant.edu

**This PDF file includes:**

Supplementary Text

Figs. S1 to S4

Table S1

Data S1 to S2 (separate file)

References (1 to 16)

Supplementary Text

Age Determination for Sampling Sites:

Previous age assignment for most of the sampling sites was initially based upon historical records and artifacts found within the structures, except for Site 8, the Milan Castle Heritage Site, where a previous radiometric age is available (Table S1). To test these archaeological-based chronologies, we performed Accelerator Mass Spectrometry (AMS) ^14^C dating on selected samples from both the eastern and western clusters. All evidence establishes a Han Dynasty age for sampling sites in the eastern cluster (Sites 1-7) along the Shule River in Gansu (See Fig. 1 in main manuscript). The most definitive evidence comes from large amounts of bamboo and wooden strips found at Great Wall sites near Dunhuang and nearby ruins with writings that constrains the age to within 230 years of the Han Dynasty, specifically between 98 BC and 137 AD^1^. Other archaeological artifacts, such as silk textiles and construction style of these beacon towers, confirm that these military structures were key stops along the ancient Silk Road during the Han Dynasty^2^. Abundant Han era literature^3^ also demonstrate that military activities were connected with these structures during the Han period. Two ^14^C AMS dates from Site 7 yielded radiometric ages of 132 and 116 BC, confirming the Han Dynasty age (Table 1, Table S1).

Ages from sampling sites in the western cluster (Sites 8-14) of Xinjiang are not as well-known. Site 8 was radiometrically dated to the Tang Dynasty^4^, while a recent radiocarbon date obtained from human patella collagen from Site 9 (Buddha Tower and burial site, Yingpan City Heritage Site) yielded an age of 1730 ± 30 BP, and calibrated to a median age of 305 AD, placing it within the Jin Dynasty (266 – 420 AD)^5^. Site 10 (Yingpan City Heritage Site) may be contemporary to Site 9 but was likely constructed during the Han Dynasty and continuously occupied through the Jin period^6^.

Recent archaeological studies unearthed abundant Tang Dynasty artifacts from the Keyakekuduke Beacon Tower site, a watchtower that belongs to the same beacon tower series as our Sites 11 and 12 along the Kongque River, suggesting a Tang Dynasty age for this cluster^7^. Our new radiometric dating confirms a Tang Dynasty age for these sites. AMS analyses on four *Phragmites* samples from Sites 11 and 12 yielded calendar years between 677 – 726 AD, dates which are consistent with both archaeological findings and historical documentation^7^.

We obtained two AMS dates from samples collected at Site 14 (Sishilidadun Beacon Tower) that yielded median ages of 1030 and 1160 AD, placing the structure to within the Song Dynasty (960 – 1279 AD). Site 13 likely dates to this period, but we did not yield any biochemical data from samples from this location.

Below, we provide a detailed site-by-site analysis for individual sampling locations with our new AMS dates compounded with evidence and references from which the above conclusions were based upon:

Site 1 (Great Wall segment near Qiaowan, Anxi County) and Site 2 (Beacon tower near Site 1). These sites were described in detail by Yue and Zhong^3^. Five-baht coins, the typical Han Dynasty currency, and Han-style pottery fragments attribute these locations to the Han Dynasty.

Site 3 (Beacon tower near Guazhou town) and Site 4 (Xijiandun Beacon Tower). The architectural styles and writing on bamboo and wood strips found nearby are typical of the Han Dynasty^2^. These towers were surveyed and described in Yue and Zhong^1^, substantiating the Han Dynasty age for these structures.

Site 5 (Cang Ting Sui Beacon Tower at Yumenguan), Site 6 (Great Wall Heritage Site), and Site 7 (the Majuanwan Great Wall segments). The age of these ruins was initially described by Stein^8^ based on more than 1,000 wood and bamboo strips. These collections, along with some additional information^1,9^, attribute these wall fragments and beacon towers to the Han Dynasty. Other artifacts, such as silk strips and fish shaped silk products, associate with the Han period^10^. Archaeological excavation at the Majuanwan site in 1979 uncovered 1,217 bamboo and wooden strips with writing that documented military, economic, law, administrative, and diplomatic policies of the Han Dynasty^11^. Our new AMS dates from two *Phragmites* samples from Site 7 yielded median ages of 132 and 116 BC, confirming the Han Dynasty assignment.

Site 8 (Milan Castle Heritage Site). Radiometric dating of organic material from the castle wall yielded a calendar age of ~770 AD, belonging to the Tang Dynasty^4^. This date is consistent with Tang architectural styles, regional symbols, and historical records of this site^12^.

Site 9 (Buddha Tower and Burial Site, Yingpan City Heritage Site). A recent radiocarbon age from extracted collagen from the patella of “Yingpan Man” buried at Site 9, yielded a date of 1730 ± 30 BP, a calibrated range of 245 to 385 AD, and a median age of 305 AD. This radiocarbon age attributes the burial site to the Jin Dynasty (266 – 420 AD)^5^.

Site 10 (City wall, Yingpan City Heritage Site). Located about one and a half kilometers from Site 9. The style of the ancient city’s architecture and associated artifacts (e.g., copper mirrors, pottery, five-baht coins), are believed to be of the Han Dynasty^6,13^. This age assignment is consistent with the style of textiles, art patterns, and writing of regional languages belonging to the Eastern Han dynasty^14^. Artifacts from burials suggest that the Yingpan City continued to be used until the Jin Dynasty, however, making it at least partially contemporary with Site 9.

Site 11 (Yakelun Beacon Tower) and Site 12 (Sunji Beacon Tower). Recent archaeological discoveries in the lower (southern) reach of the Kongque River uncovered abundant Tang Dynasty (618 – 907 AD) artifacts associated at the Keyakekuduke beacon tower, suggesting that they were garrisoned during the Tang period^7^. We obtained AMS ages from four *Phragmites* samples from Sites 11 and 12 that date to 677 – 726 AD, confirming the Tang Dynasty age of this cluster.

Site 13 (Tahaqi Beacon Tower) and Site 14 (Sishilidadun Beacon Tower). These two sites are located north of the Bosten Lake and do not belong to the series of beacon towers along the Kongque River to the south. Surveys and preliminary studies by the Archeological Bureau of Xinjiang Uygur Autonomous Region^15,16^ assigned them to the Han Dynasty based upon the architectural styles of these beacon towers. However, our new AMS ages obtained from Site 14 indicate that material used to construct the tower date to 1030 – 1160 AD. Therefore, these structures were built during the Song Dynasty, an era in which Chinese political borders did not extend into the Tarim Basin. As the eastern Tarim Basin was within the Western Liao Dynasty at this time, it is possible Sites 13 and 14 were defensive styles imported from northeastern China when remnant Liao loyalists established the Western Liao in Central Asia.

Additional Nonparametric Statistical Tests:

In addition to the two-tailed Student’s t-Tests and Mann-Whitney *U* tests used to examine the significance in differences between sample sets presented in the main manuscript, we also performed Spearman’s rank tests to correlate geographical variables and δ^13^C and δ^15^N values and Kruskal-Wallis tests to compare δ^13^C and δ^15^N values across ancient and modern sample sets using PAST 4.03. There is a significant difference between δ^13^C values for all sites and ages (Kruskal-Wallis Test, *p*=0.001), which is largely attributed to the heavy mean δ^13^C value of modern *P. australis* from the western cluster (Table S2, top/right). However, there is also a difference between Jin and Han samples (Kruskal-Wallis Test, *p*=0.010), likely due to Jin samples being ~0.8 ‰ lighter than Han-aged *Phragmites*. This further suggest a differential rate of environmental change on opposite sides of Lop Nur as there is no significant different between ancient Phragmites and living P. australis from the eastern cluster (Kruskal-Wallis Test, *p*=0.843) (also see Fig. 8 in main manuscript). There is also a significant difference between δ^15^N values for all sites and ages (Kruskal-Wallis Test, *p*=0.009), which is attributed to the heavy mean δ^15^N values from Tang and Song sample sets (Table S2, bottom/left). Unfortunately, we do not have δ^15^N of living *P. australis* from the eastern cluster.

Longitude is a strong geographical predictor of δ^13^C values when all ancient *Phragmites* (Spearman’s correlation, *r_s_*=0.343, *p*=0.026) and all modern *P. australis* (Spearman’s correlation, *r_s_*=-0.699, *p*=0.011) are considered (Table S3.1). However, there are no significant correlations when all ages are considered separately, but this is expected given the small geographic range of each site. Nitrogen, on the other hand, does show significant correlations when individual ages and sites are examined, for example, Jin (Spearman’s correlation, *r_s_*=0.866, *p*=0.200) and Tang (Spearman’s correlation, *r_s_*=0.756, *p*=0.043) sites (Table S3.2). This needs to be investigated further with additional δ^15^N measurements on both living *P. australis* and Great Wall *Phragmites*, however.

References

1. Z.-y. Luo, G.-w. Wang, *Lost strips in sands (流沙坠简) (in Chinese)*. (Chung Hwa Book Company, Shanghai, China, 1993).

2. B. Li, An investigation and study on the post system of the Han Dynasty and the places and ruins of Zhi and Qizhi and Yi in Dunhuang. *Dunhuang Research* **127**, 70-77 (2011).

3. B. Yue, S. Zhong, *Survey report of Han Dynasty Great Walls along the Shule River (in Chinese)*. B. Yue, S. Zhong, Eds., (Wenwu Press, Beijing, 2001), pp. 254.

4. H. Lü et al., A preliminary study of chronology for a newly-discovered ancient city and five archaeological sites in Lop Nor, China. *Chinese Science Bulletin* **55**, 63-71 (2010).

5. T. Wang et al., Revealing lost secrets about Yingpan Man and the Silk Road. *Scientific Reports* **12**, 669 (2022).

6. Xinjing Institute of Archeology, Archeological report on Yingpan #15 burial site in Yuli, Xiangjiang (in Chinese with English summary). *Cultural Relics* **1**, 4-16 (1999).

7. Archeological Bureau of Xinjing Uygur Autonomous Region, The Tang Dynasty Keyakekuduke Watchtower site in Yuli County of Xinjiang Province. *Kaogu* *8*, 23-44 (2021).

8. M. A. Stein, "Serindia Detailed Report of Explorations in Central Asia and Westernmost China," *Digital Silk Road* (1921).

9. É. Chavannes, *Les documents chinois découverts par Aurel Stein dans les sable du Turkestan oriental*. (Oxford Press, Oxford, UK, 1913).

10. L. Wang, From garrison reclamation to border defense – taking Yumen Pass as an example to discuss the economic and military factors of the Han Dynasty Great Wall system (in Chinese with English abstract). *Journal of Human Settlements in West China* **32**, 40-46 (2017).

11. Gansu Museum and Dunhuang Cultural Institute, Archeological report of Maquanwan Han Dynasty beacon tower site near Dunhuang. *Cultural Relics* **10**, 1-8 (1981).

12. Z. Wang, *Evidence of Tubo Transmission in the New Tang Book (in Chinese)*. (Science Press, 1958).

13. J.-L. Zhou, On the archeology of Yingpan burial sites in Yuli County, Xinjiang. *The Western Regions Studies* **3**, 59-66 (1999).

14. F. Zhao, *Silks from the Silk Road: Origin, transmission, and exchange (in Chinese)*. (Zhejiang University Press, Hangzhou, China, 2015).

15. Archeological Bureau of Xinjing Uygur Autonomous Region, *Unmoveable Artifacts in Bayingolin Mongol Autonomous Prefecture (in Chinese)*. (Xinjiang Arts and Photograph Press, 2015).

16. Archeological Bureau of Xinjing Uygur Autonomous Region, "Report on survey of Great Wall resources in the Xinjing Uygur autonomous region (in Chinese)," (2014).


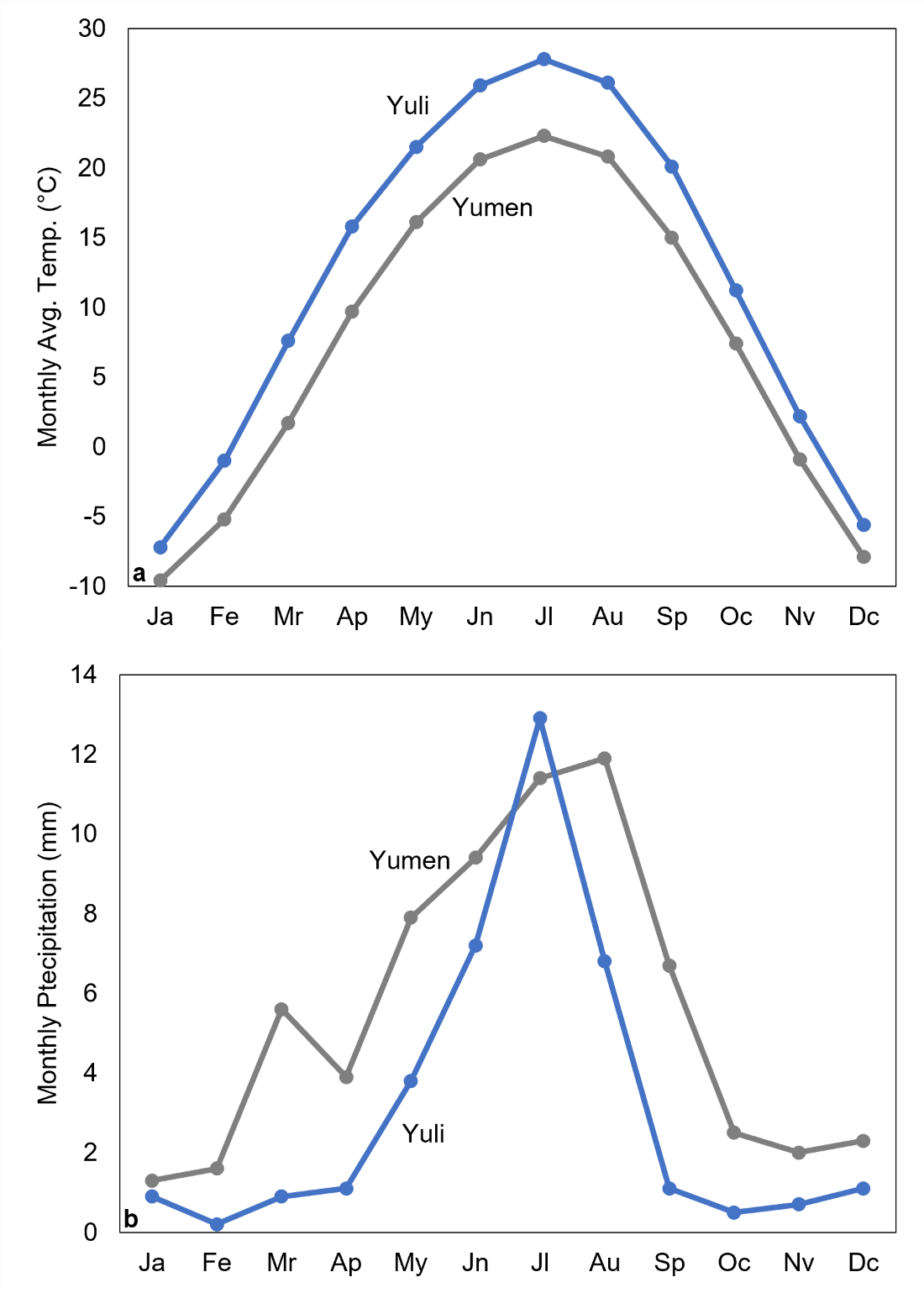


Fig. S1. Modern climate parameters representing the eastern (grey) and western (blue) cluster samples. Monthly average temperature (a) and precipitation (b) data from Yumen, Gansu (40°16' N, 97°2' E) and Yuli, Xinjiang (41°21' N, 86°16' E).


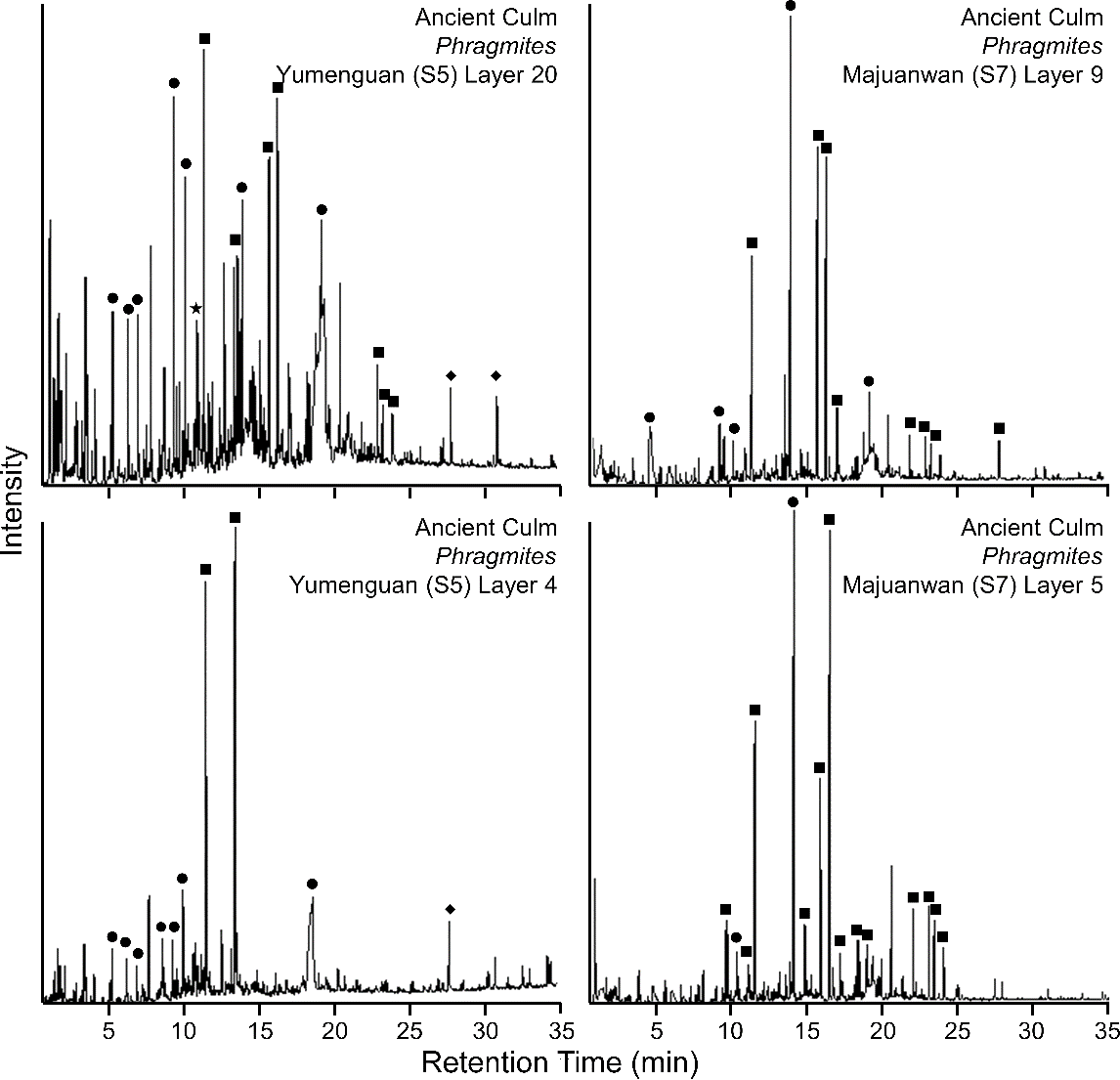


Fig. S2. Additional partial ion chromatograms. The Py-GC-MS analysis of ancient *Phragmites* culms from different wall fascines and the distribution of ● Polysaccharide, ■ Lignin, ♦ Fatty Acid, and ★ Amino Acid. The “S,” as in S7, stands for “Site.” See Data S2 for compound identifications.


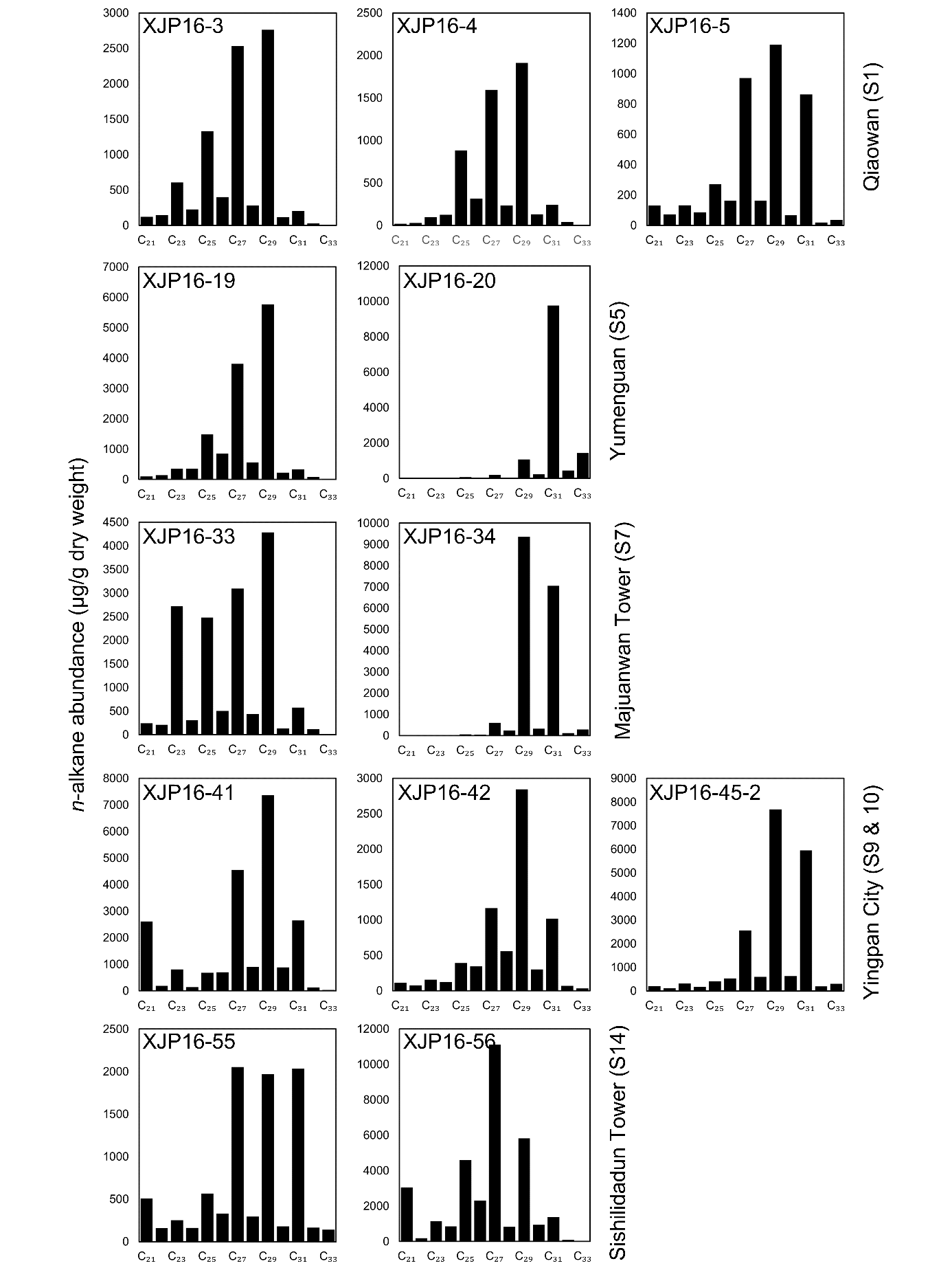


Fig. S3. C_21_-C_33_ *n*-alkane abundances. Distribution of *n*-alkanes by chain length and their abundances in μg/g of dry weight for modern *P. australis* samples. Refer to Figure 1 and Data S1 for sample location and designation.


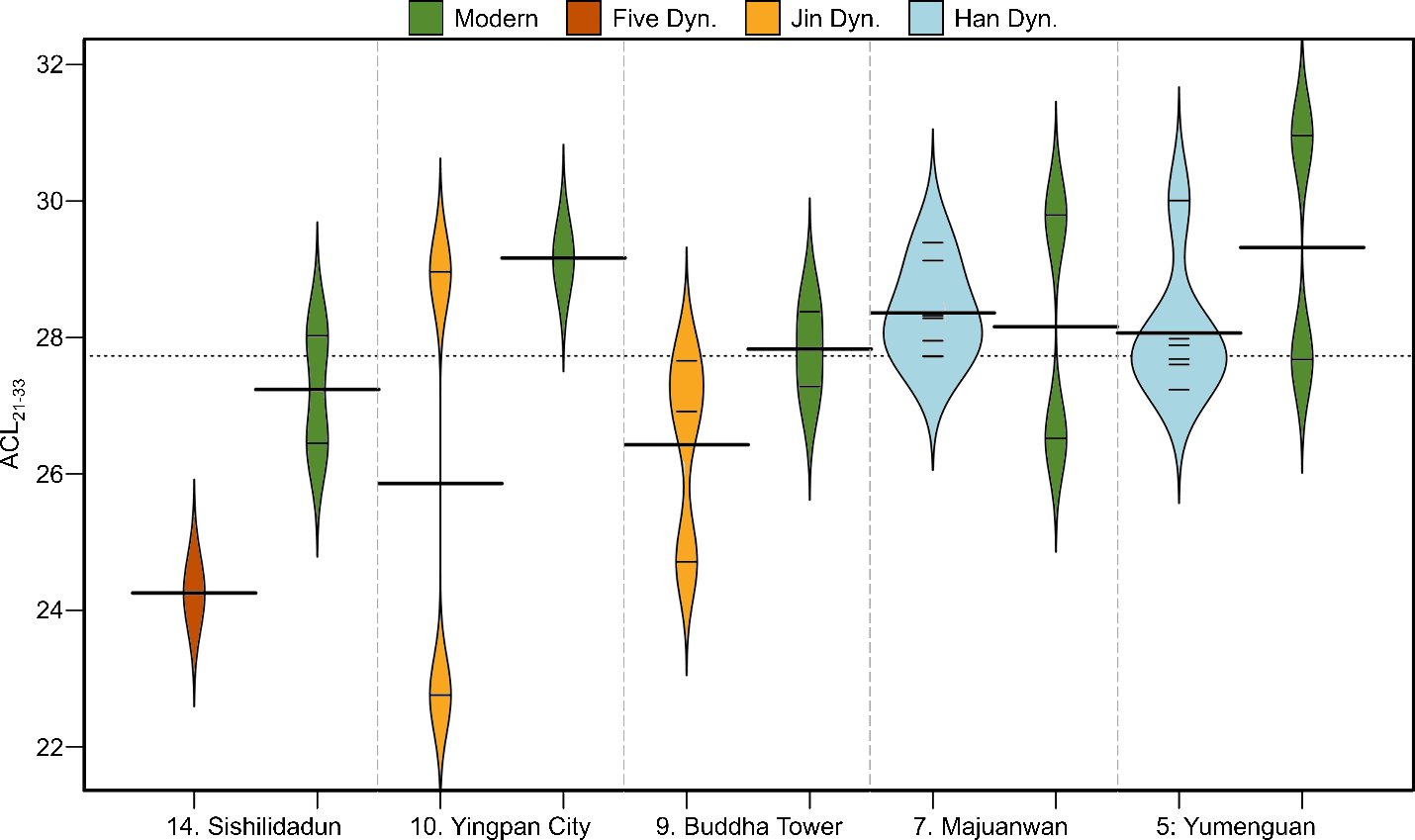


Fig. S4. ACL bean plots by site. Average chain length of the C_21_-C_33_ *n*-alkanes (ACL_21-33_) separated into sites that had both modern *P. australis* and ancient *Phragmites* samples analyzed. The thick black lines show the mean value from each period, while the thin black lines represent individual data points. The shape of each polygon represents the estimated density of the data, and the dashed line is the mean of all samples.

Table S1. Summary of Sites, Dynastic Periods, Dating Methods, and References.

| **Site** | **Dynasty** | **Technique/Evidence** | **Reference** |
| --- | --- | --- | --- |
| 1: Han Dynasty Great Wall segment near Qiaowan, Anxi County | Han | Archaeology/Coins, Pottery | 3 |
| 2: Beacon tower near Site 1 | Han | Archaeology/Coins, Pottery | 3 |
| 3: Beacon tower near Guazhou town | Han | Archaeology/Architecture, wood and bamboo strips | 2, 3 |
| 4: Xijiandun Beacon Tower | Han | Archaeology/Architecture, wood and bamboo strips | 2, 3 |
| 5: Cang Ting Sui Beacon Tower at Yumenguan | Han | Archaeology/Wood and bamboo strips, silk products | 1, 8, 9, 10 |
| 6: Great Wall Heritage Site | Han | Archaeology/Wood and bamboo strips, silk products | 1, 8, 9, 10 |
| 7: Majuanwan Great Wall segments | Han | New AMS dating and archaeology/Wood and bamboo strips, silk products | 1, 8, 9, 10 |
| 8: Milan Castle Heritage Site | Tang | Radiometric Dating | 4, 12 |
| 9: Buddha Tower, Yingpan City Heritage Site | Eastern Han | Archaeology/Burial style and associated artifacts | 5, 6, 13, 14 |
| 10: City wall, Yingpan City Heritage Site | Eastern Han | Archaeology/Burial style and associated artifacts | 6, 13, 14 |
| 11: Yakelun Beacon Tower | Tang | New AMS dating and archaeology/Artifacts from nearby beacon tower | 7, this study |
| 12: Sunji Beacon Tower | Tang | New AMS dating and archaeology/Artifacts from nearby beacon tower | 7, this study |
| 13: Tahaqi Beacon Tower | Song | Archaeology/Artifacts from nearby beacon tower | 7 |
| 14: Sishilidadun Beacon Tower | Song | New AMS dating and archaeology/Artifacts from nearby beacon tower | 7, this study |

**Table S2.** Kruskal-Wallis Test *p*-value results for δ^13^C (top/right) and δ^15^N (bottom/left) for ancient and modern *Phragmites*.

|  | **Han** | **Jin** | **Tang** | **Song** | **Mod. East** | **Mod. West** |
| --- | --- | --- | --- | --- | --- | --- |
| **Han** | - | 0.010 | 0.223 | 0.197 | 0.843 | <0.001 |
| **Jin** | 0.788 | - | 0.164 | 0.456 | 0.088 | 0.009 |
| **Tang** | 0.008 | 0.028 | - | 0.307 | 0.488 | 0.003 |
| **Song** | 0.010 | 0.025 | 0.838 | - | 0.210 | 0.025 |
| **Mod. East** | n/a | n/a | n/a | n/a | - | 0.004 |
| **Mod. West** | 0.283 | 0.456 | 0.153 | 0.127 |  | - |

**Table S3.1** Spearman’s rank correlations for longitude and δ^13^C of ancient and modern *Phragmites*.

|  | **No. Samples** | **^1^No. of Sites** | **Mean δ^13^C** | **Coefficient (*r_s_*)** | ***p*-value** |
| --- | --- | --- | --- | --- | --- |
| **All Ancient** | 42 | 13 | -23.9 ± 0.7 | 0.343 | 0.026 |
| **All Modern** | 12 | 6 | -22.9 ± 1.3 | -0.699 | 0.011 |
| **Han** | 26 | 7 | -23.7 ± 0.6 | 0.117 | 0.568 |
| **Jin** | 5 | 2 | -24.5 ± 0.5 | -0.289 | 0.800 |
| **Tang** | 8 | 3 | -24.0 ± 0.6 | -0.176 | 0.679 |
| **Song** | 3 | 1 | -24.5 ± 1.2 | n/a | n/a |
| **Mod. East** | 7 | 3 | -23.7 ± 0.8 | 0.189 | 0.686 |
| **Mod. West** | 5 | 3 | -21.7 ± 0.7 | 0.800 | 0.800 |

**Table S3.2** Spearman’s rank correlations for longitude and δ^15^N of ancient and modern *Phragmites*.

|  | **No. Samples** | **^1^No. of Sites** | **Mean δ^15^N** | **Coefficient (*r_s_*)** | ***p*-value** |
| --- | --- | --- | --- | --- | --- |
| **All Ancient** | 42 | 13 | 9.3 ± 6.7 | -0.211 | 0.181 |
| **All Modern** | 3 | 2 | 4.6 ± 7.6 | n/a | n/a |
| **Han** | 26 | 7 | 6.9 ± 3.6 | 0.259 | 0.201 |
| **Jin** | 5 | 2 | 6.1 ± 3.2 | 0.866 | 0.200 |
| **Tang** | 8 | 3 | 16.3 ± 10.4 | 0.756 | 0.043 |
| **Song** | 3 | 1 | 15.9 ± 4.0 | n/a | n/a |
| **Mod. East** | 0 | 0 | n/a | n/a | n/a |
| **Mod. West** | 3 | 2 | 4.6 ± 7.6 | 0.866 | 0.667 |

^1^Refers to the number of distinct sampling locations that have longitude and latitude GPS coordinates (see Data S1).

Data S1. (separate file)

Data S1. Sample age, location information, and biochemical data for modern *P. australis* and ancient *Phragmites* collected at 14 sites in northwestern China.

Data S2. (separate file)

List of compound identification and inferred origin of the Py-GC-MS analyses for modern *P. australis* and ancient *Phragmites*.
